# A Secretory Protein Laccase lac8 From Pathogenic Fungi Activates Plant Protein 14‐3‐3 and Leucine‐Rich Repeat Receptor‐Like Protein LRR‐RLP1 to Trigger Mango Immunity

**DOI:** 10.1111/mpp.70163

**Published:** 2025-10-29

**Authors:** Mengting Zhang, Jinji Pu, Chen Lei, Min Zhu, Aiping Gao, Yeyuan Chen, He Zhang

**Affiliations:** ^1^ National Key Laboratory for Tropical Crop Breeding, Key Laboratory of Integrated Pest Management on Tropical Crops, Ministry of Agriculture and Rural Affairs Chinese Academy of Tropical Agricultural Sciences Environment and Plant Protection Institute Haikou China; ^2^ College of Tropical Agriculture and Forestry Hainan University Haikou China; ^3^ Laboratory of Crop Gene Resources and Germplasm Enhancement in Southern China, Ministry of Agriculture and Rural Affairs Chinese Academy of Tropical Agricultural Sciences Tropical Crops Genetic Resources Institute Haikou China; ^4^ Sanya Research Institute of Chinese Academy of Tropical Agricultural Sciences Sanya China

**Keywords:** 14‐3‐3 protein, *Colletotrichum gloeosporioides*, laccase, leucine‐rich repeat receptor‐like protein, mango, plant immunity

## Abstract

Plants cope with the biotic stress caused by pathogen infection through complex resistance mechanisms. Here, we identified a secreted laccase Cglac8 from *Colletotrichum gloeosporioides* and confirmed its involvement in *C. gloeosporioides* infection of mango (*Mangifera indica*) plants, as well as its ability to activate the host's innate immune mechanism. Cglac8 interacted with MiLRR‐RLP1 and Mi14‐3‐3‐D1 as demonstrated by yeast two‐hybrid, bimolecular fluorescence complementation and pull‐down assays. The interacting proteins MiLRR‐RLP1 and Mi14‐3‐3‐D1 positively regulate mango resistance to *C. gloeosporioides* by promoting the reactive oxygen species burst and biosynthesis of phytohormones. When Cglac8, MiLRR‐RLP1 and Mi14‐3‐3‐D1 proteins were overexpressed together in mango, the resistance of mango to *C. gloeosporioides* was significantly enhanced. Our findings reveal a new defence mechanism of host plants against *C. gloeosporioides*, providing a theoretical basis for disease‐resistant molecular breeding. The dual role of secretory laccase Cglac8 may reflect a balancing mechanism in host–pathogen co‐evolution.

## Graphical abstract

A secreted laccase Cglac8 from *Colletotrichum gloeosporioides* targets host mango protein 14‐3‐3 and leucine‐rich repeat receptor‐like protein LRR‐RLP1 to trigger an immune response by promoting the biosynthesis of phytohormones.

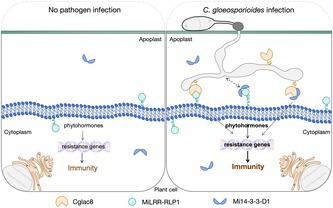

## 1 Introduction

Pathogen infection is one of the greatest threats to the growth and development of plants in nature (Hu et al. 2022). To cope with the biotic stresses caused by pathogens, plants have evolved multiple complex and effective resistance mechanisms (Atkinson and Urwin 2012; Engelsdorf and Hamann 2014). One of the most important factors for plant adaptation is the perception of microbial‐associated molecular patterns (MAMPs), and the activation of the corresponding immune response against pathogens (Boller and Felix 2009).

Plants have evolved cell‐surface pattern recognition receptors (PRRs) to detect MAMPs, thereby activating pattern‐triggered immunity (PTI) (Jones and Dangl 2006). Receptor‐like kinases (RLKs) and receptor‐like proteins (RLPs), belonging to the PRR family, are involved in plant defence and development (Jones et al. 1994; Nadeau and Sack 2002). RLKs are mainly involved in regulated recognition and signal transduction (Kim et al. 2024; Boutrot and Zipfel 2017). Unlike RLKs, the molecular mechanisms of RLP recognition and signal transduction are still poorly understood (Hohmann et al. 2017). Previous studies have shown that RLPs lack intracellular kinase domains (Macho and Zipfel 2014) and need to interact with other proteins containing kinase domains, such as SOBIR1 (Liebrand, van den Berg, et al. 2013), to activate downstream signalling (Liebrand, van den Burg, and Joosten 2013). Leucine‐rich repeat receptor‐like proteins (LRR‐RLPs) usually use the LRR domain to recognise invading molecules (Kim et al. 2024) and thus participate in the activation of immune responses (Fritz‐Laylin et al. 2005). For example, LRR‐RLP RXEG1 recognises XEG1 of *Phytophthora sojae* in the apoplast through the LRR domain, then forms a complex with LRR receptor‐like kinases BAK1 and SOBIR1 to transduce XEG1‐induced defence signals in *Nicotiana benthamiana* (Sun et al. 2022). AtRLP23 mediates the recognition of a peptide motif of necrosis and ethylene‐inducing peptide 1‐like proteins (NLPs) from many bacterial, fungal and oomycete (Albert et al. 2019). In *Arabidopsis thaliana* , *Oryza sativa* and *Solanum lycopersicum* , LRR‐RLK SOBIRs individually bind to LRR‐RLPs (ReMax, OsRLP1 and Cf‐4), and these RLK/RLP complexes mediate immune responses (Liebrand, van den Berg, et al. 2013; Zhang et al. 2021; Jehle et al. 2013). However, the identification and molecular mechanism of LRR‐RLPs in mango (*Mangifera indica*) remain unclear. LRRs not only act as a key regulator of plant immunity but also of growth and development (Chinchilla et al. 2009; Liebrand, van den Burg, and Joosten 2013; De Smet et al. 2009). AtSERK1, as an LRR‐RLK protein, mediates embryo development via interaction with phosphatase AtCDC48, a member of the *Arabidopsis* 14‐3‐3 protein family (Rienties et al. 2005). 14‐3‐3 protein serves as the immune centre (Obsilova and Obsil 2022; Zhao et al. 2021) and scaffold protein (Zhang et al. 2024) of plants and is targeted by pathogen effectors (Nomura et al. 2006; Lozano‐Durán et al. 2014; Li et al. 2013; Seo et al. 2023). It enhances disease resistance in the plant cell (Konagaya et al. 2004; Dong et al. 2023) but has also been found to play a function outside the plant cell (Wen et al. 2007; Voigt and Frank 2003). Some studies have found that the components of nucleotide‐binding leucine‐rich repeat immune receptor complexes can interact with 14‐3‐3 protein, activating mitogen‐activated protein kinases and effector‐triggered immunity (Sheikh et al. 2023).

Plant diseases caused by fungi (Flores‐Nunez and Stukenbrock 2024), such as wheat stem rust caused by *Puccinia graminis* f. sp. *tritici* (Cheng et al. 2017) and tomato leaf mould caused by *Cladosporium fulvum* (Rooney et al. 2005), seriously threaten food security and disturb the natural ecosystem (Franceschetti et al. 2017). Filamentous pathogens secrete effectors during host colonisation to suppress host immunity and cause disease (Kanja and Hammond‐Kosack 2020; Wang and Wang 2018). For example, *P. graminis* f. sp. *tritici* PSTha5a23 suppresses PTI via inhibition of callose deposition and programmed cell death (Cheng et al. 2017), and *C. fulvum* Avr2 inhibits Cys proteases and induced hypersensitive response (HR) in tomato lines that carry the cognate R protein Cf‐2 (Rooney et al. 2005). *C. gloeosporioides* can secrete enzymes (laccase, pectinase, cellulase, etc.) and other substances to degrade the cell wall during invasion (Mehta and Baghela 2021; Yu et al. 2022). Laccase (EC 1.10.3.2) is an oxidoreductase with polyphenol oxidase activity; it acts as an important virulence factor of fungi, and often exists in the form of a multigene family. Fungal laccase is involved in the decomposition of lignocellulosic polymers, defence/protection, virulence, pathogenic mechanism, pigmentation and sporulation (Liu et al. 2020). During the pathogenic process, it can damage the plant cell wall, oxidise phenolic and non‐phenolic substances produced by the host, and promote the colonisation by pathogens (Liu et al. 2020). It has diverse roles in different plant‐pathogenic fungi and even the same pathogen (Janusz et al. 2020). The roles of laccase in *C. gloeosporioides* remain largely obscure.

Here, we identified Cglac8, a secreted laccase from *C. gloeosporioides*, as an important pathogenicity factor of *C. gloeosporioides* and an elicitor of innate immunity. We also demonstrated that Cglac8, MiLRR‐RLP1 and Mi14‐3‐3‐D1 could interact with each other, and that the protein complex of MiLRR‐RLP1 and Mi14‐3‐3‐D1 mediated plant resistance to *C. gloeosporioides*. Our work provides possibilities and a theoretical basis for designing new pesticide targets and molecular breeding of mango disease resistance.

## 2 Results

### 2.1 *C. gloeosporioides Cglac8* Is a Key Pathogenicity Gene Encoding a Secreted Protein

**FIGURE 1:**
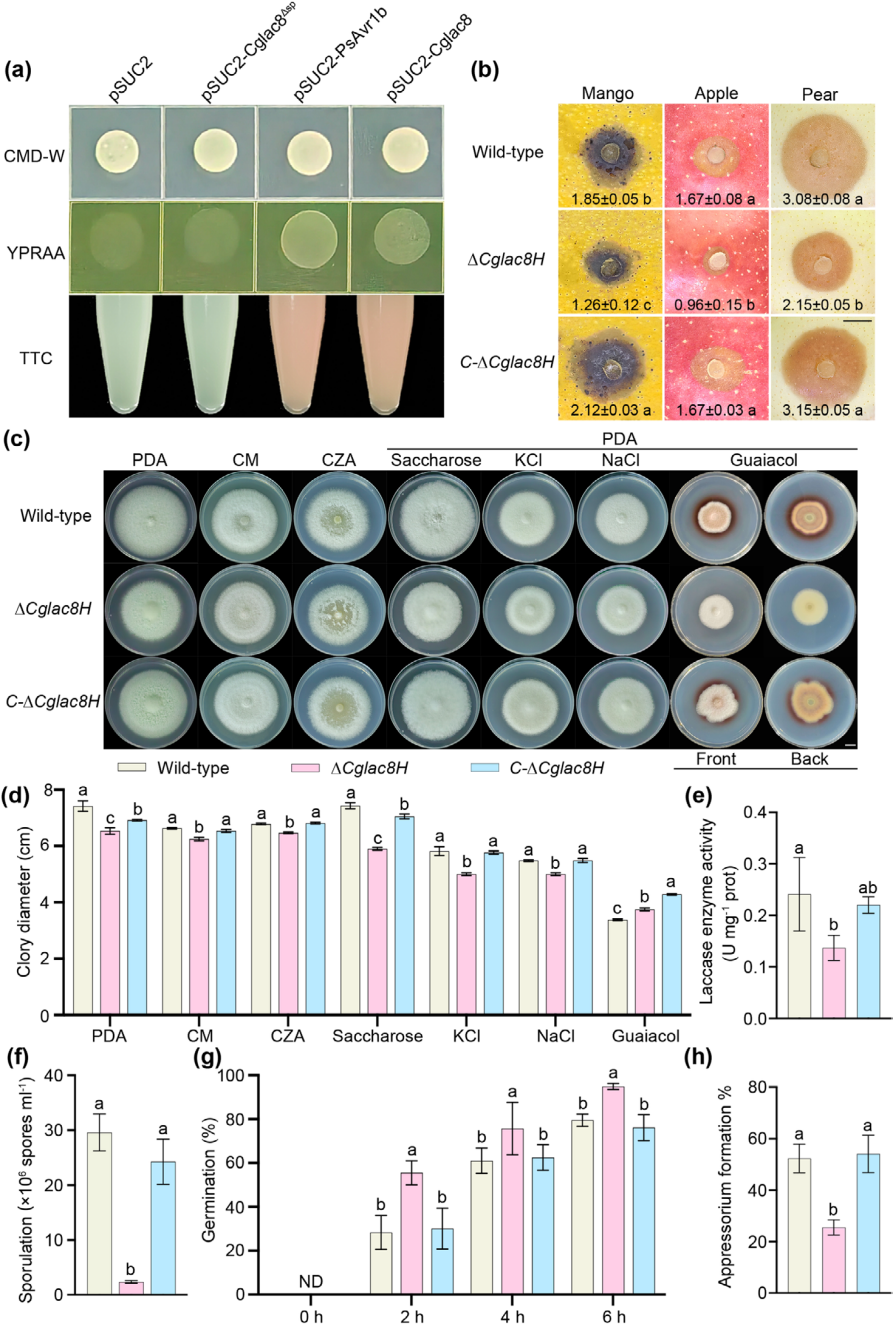
*Cglac8* acts as an important pathogenicity factor of *Colletotrichum gloeosporioides*. (a) Verification of Cglac8 secretion. The yeast carrying pSUC2‐Cglac8 and pSUC2‐Cglac8^∆SP^ was grown on CMD−W and YPRAA media, and the 2,3,5‐triphenyltetrazolide chloride (TTC) reduction colour reaction was performed. The empty vector (pSUC2) was used as the negative control. pSUC2‐PsAvr1b was used as the positive control. (b) Pathogenicity of *Cglac8*. The wild‐type, ∆*Cglac8H* and *C‐*∆*Cglac8H* were inoculated on wounded mango, apple and pear fruits at 28°C. After 5 days post‐inoculation, the symptoms were observed and the lesion area was measured. (c, d) Colony morphology (c) and diameter (d) of different strains. The wild‐type, ∆*Cglac8H* and *C‐*∆*Cglac8H* grown on potato dextrose agar (PDA), complete medium (CM), Czapek‐Dox agar (CZA), PDA containing 30 g L^−1^ saccharose, 1 M KCl, 1 M NaCl or 0.04% guaiacol for 5 days at 28°C. (e) The quantitative determination of fungal laccase activity of the Cglac8 of *C. gloeosporioides*. (f) The number of conidia. The conidial production of wild‐type, ∆*Cglac8H* and *C‐*∆*Cglac8H* was counted after cultivation for 3 days at 28°C with shaking. (g) Conidial germination rates of wild‐type, ∆*Cglac8H* and *C‐*∆*Cglac8H*. The number of germinated conidia was counted after 0, 2, 4 and 6 h. ND is not detected. (h) The penetration rate of appressoria at 6 h. Data are means ± SD from 3 or more biological replicates. Different lowercase letters indicate significant differences at *p* < 0.05 (one‐way ANOVA).

In preliminary experiments, we found that the laccase transcription level of *Cglac8* was distinctly induced during *C. gloeosporioides* infection of mango (Figure S1), suggesting that *Cglac8* may play an important role in the interaction between *C. gloeosporioides* and mango. Further bioinformatics analysis showed that Cglac8 encodes a 591‐amino‐acid protein containing a signal peptide (SP) and three copper (Cu) oxidase Pfam motifs (Figure S2). To investigate whether *Cglac8* is involved in the infection process of *C. gloeosporioides*, we first validated the secretion of Cglac8 via a yeast expression system. Yeast carrying pSUC2‐Cglac8 showed normal growth on YPRAA medium, similar to the positive control pSUC2‐PsAVr1b, whereas the pSUC2‐Cglac8^∆SP^ and the negative control pSUC2 failed to grow (Figure 1a). Moreover, the results of 2,3,5‐triphenyltetrazolide chloride (TTC) colour reaction were consistent with the growth on YPRAA medium (Figure 1a). The results suggest that Cglac8 is a secreted protein and the SP of Cglac8 serves as a functional secretion signal.

To assess the function of *Cglac8* during *C. gloeosporioides* infection, we constructed a *Cglac8* knockout mutant (∆*Cglac8H*) and the complemented strain (*C‐*∆*Cglac8H*) via polyethylene glycol (PEG)‐mediated protoplast transformation and CRISPR/Cas9‐mediated homology‐directed repair techniques, respectively (Figure S3). Subsequently, the pathogenicity of ∆*Cglac8H* and *C‐*∆*Cglac8H* were investigated on mango, apple and pear fruits. The results showed that ∆*Cglac8H* induced a significantly smaller lesion area compared with the wild type and *C‐*∆*Cglac8H* in the different fruits (Figure 1b). Notably, the *C‐*∆*Cglac8H* induced no significantly different symptom in apple and pear fruits compared with the wild type (Figure 1b). The results indicated that *Cglac8* acts as the critical factor for *C. gloeosporioides* infection. A vegetative growth assay showed that knockout of *Cglac8* caused a significant reduction in *C. gloeosporioides* mycelial growth (Figure 1c,d). In addition, wild‐type and *C‐*∆*Cglac8H* produced red circles on medium containing 0.04% guaiacol, while ∆*Cglac8H* did not produce a red circle after cultivation for 5 days, indicating ∆*Cglac8H* could not oxidise guaiacol. Quantification of the laccase activity indicated that in the *Cglac8* gene knockout mutant activity decreased by 37.8% (Figure 1e). The laccase activity of Cglac8 in the wild type was 0.026 (ΔA_420_/min/mg protein) by the ABTS method. Moreover, ∆*Cglac8H* exhibited lower sporulation and higher germination than the wild type (Figure 1f,g). The knockout of *Cglac8* also reduced the penetration efficiency of appressoria (Figure 1h). Microscopic observation of the entire process of conidial germination revealed that knockout of *Cglac8* delayed the formation of appressoria. These results suggest that Cglac8 protein has secretory properties and is an important pathogenicity factor for *C. gloeosporioides*.

### 2.2 Subcellular Localisation of Cglac8 and Activation of Host Immune Response

**FIGURE 2:**
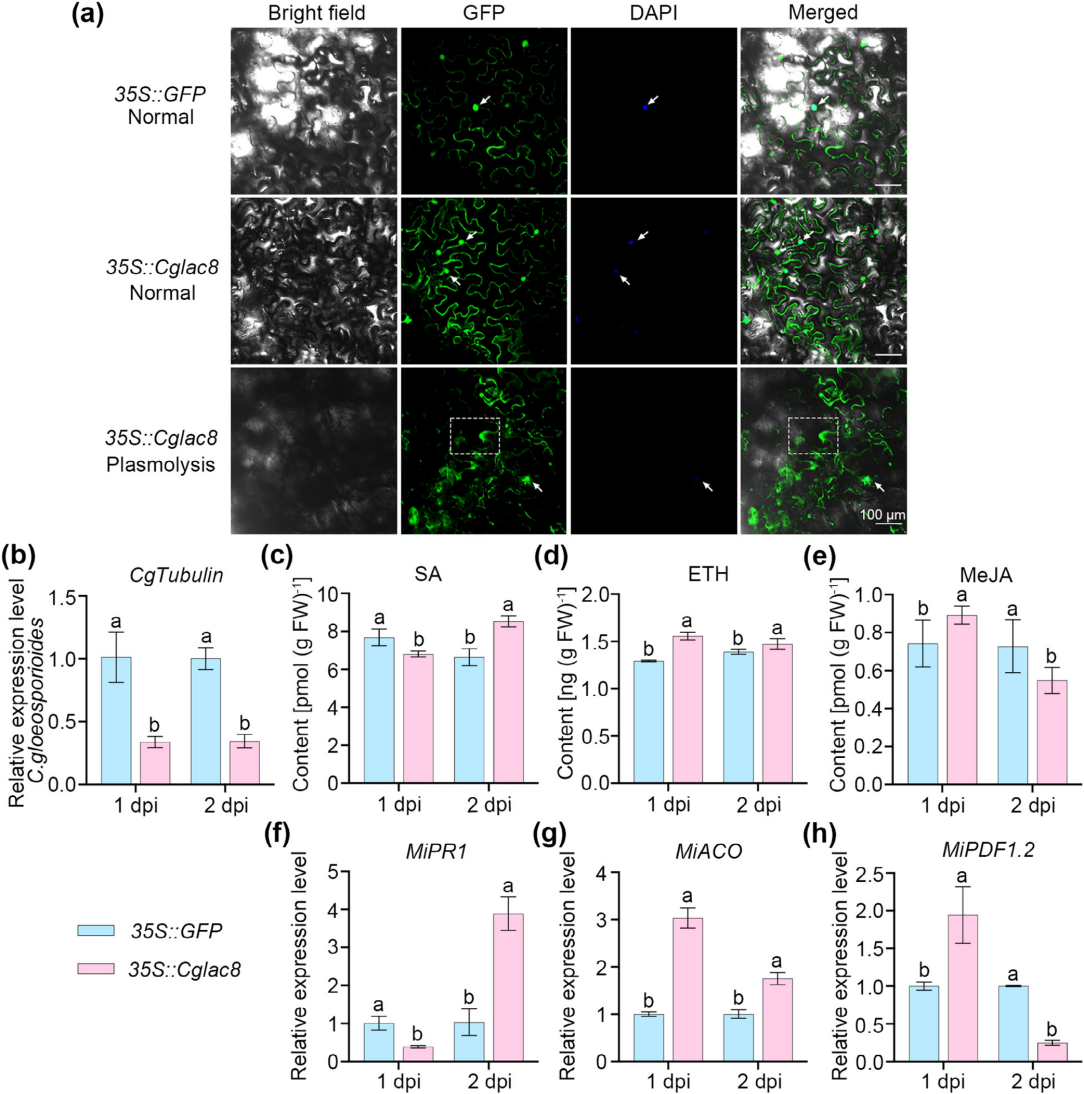
Cglac8 actives mango immunity. (a) Subcellular localisation of Cglac8. *Agrobacterium tumefaciens* GV3101 carrying the gene for the target protein was injected into *Nicotiana benthamiana* leaves for 3 days, then the leaves were stained with 4′,6‐diamidino‐2′‐phenylindole (DAPI) and used for fluorescence observation. Bars = 100 μm. The white arrow indicates the nucleus, the dashed box shows the apoplast space formed after plasmolysis. (b) The estimation of *Colletotrichum gloeosporioides* biomass in mango leaves. (c–e) The content of salicylic acid (SA) (c), ethylene (ETH) (d) and methyl jasmonate (MeJA) (e) in mango leaves after infection with *C. gloeosporioides*. (f–h) The transcript level of genes related to SA, ETH and MeJA synthesis and signal transduction in mango leaves. After confirmation that *Cglac8* was transiently overexpressed in the mango leaves, *C. gloeosporioides* was inoculated into the leaves for functional analysis. Different lowercase letters indicate significant differences at *p* < 0.05 (one‐way ANOVA). dpi, days post‐inoculation.

To further investigate the role of Cglac8 as a secretory protein, the subcellular localisation of Cglac8 was examined by confocal microscopy. In normal conditions, the expression of Cglac8 was observed in both the cytoplasm and nucleus (Figure 2a). Plasmolysis showed that the green fluorescence was found in the apoplast and nucleus (Figure 2a). These results indicate that Cglac8 is localised in the cytoplasm and nucleus, and could be secreted from the cytoplasm into the extracellular space. To further explore the involvement of *Cglac8* in the plant's immune response, *Cglac8* was overexpressed in mango leaves (Figure S4), then the leaves were infected with *C. gloeosporioides*. The *Cglac8*‐overexpressing leaves exhibited lower *C. gloeosporioides* biomass than the control leaves (Figure 2b). To explore the mechanism of *Cglac8‐*mediated mango resistance to *C. gloeosporioides*, we analysed the immune‐related hormones. The leaves overexpressing *Cglac8* exhibited higher levels of salicylic acid (SA) and ethylene (ETH) compared with those of the control at 2 days post‐inoculation (dpi) (Figure 2c,d), while methyl jasmonate (MeJA) exhibited a significantly higher level compared with the control at 1 dpi (Figure 2e). Consistently, the expression of SA, ETH and MeJA synthesis and signalling genes showed the same trend as the phytohormones level (Figure 2f–h). In summary, these results indicate that *Cglac8* activates host immunity in *C. gloeosporioides* by modulating phytohormones.

### 2.3 Cglac8 Interacts With MiLRR‐RLP1 and Mi14‐3‐3‐D1

**FIGURE 3:**
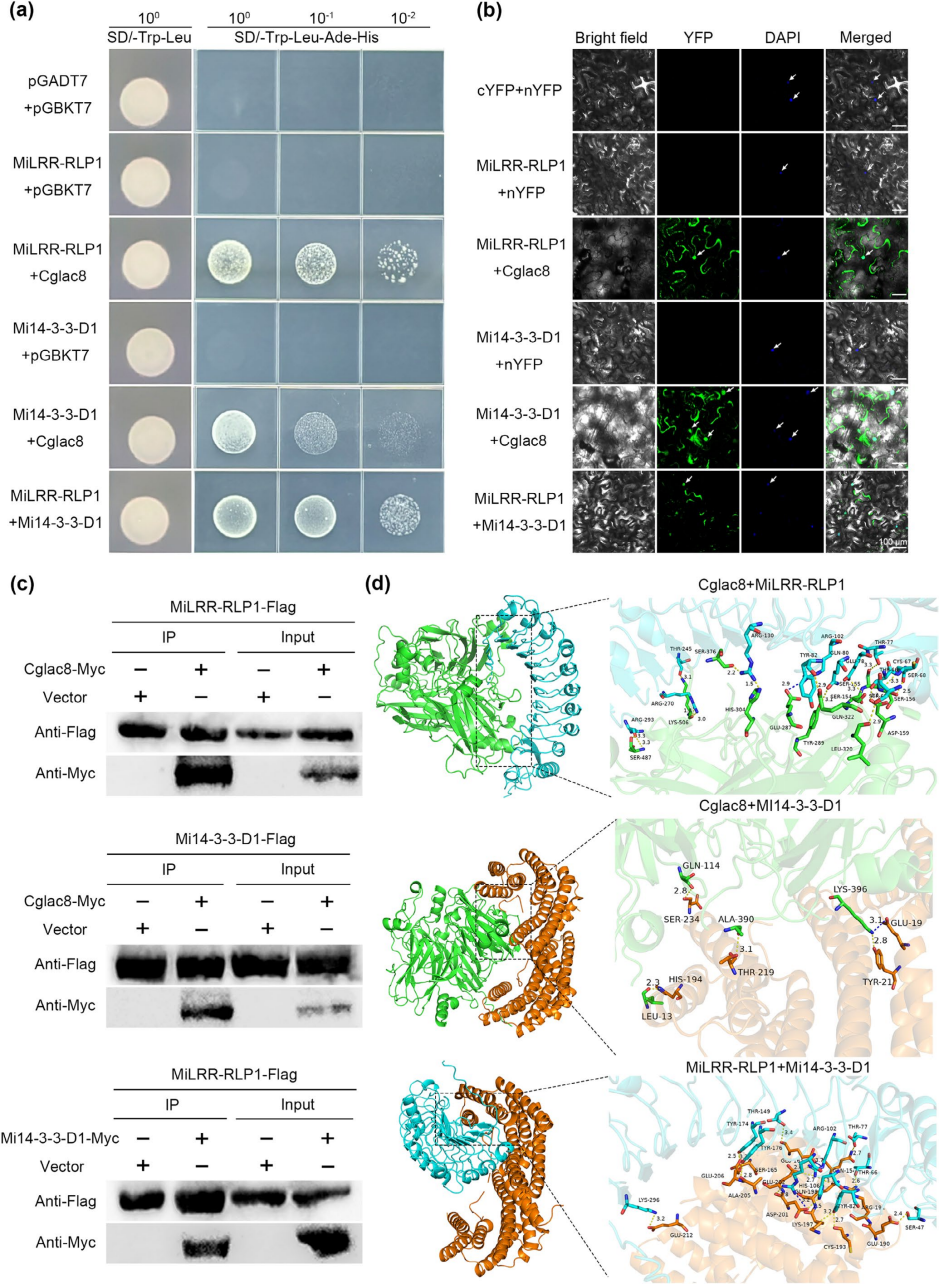
Cglac8, MiLRR‐RLP1 and Mi14‐3‐3‐D1 interact with each other in vivo and in vitro. (a–d) Yeast two‐hybrid (Y2H) assay (a), bimolecular fluorescence complementation (BiFC) assay (b), pull‐down assay (c) and molecular docking (d) showing the interaction between Cglac8, MiLRR‐RLP1 and Mi14‐3‐3‐D1. For the Y2H assay, co‐expression of MiLRR‐RLP1‐pGADT7 with Cglac8‐pGBKT7, Mi14‐3‐3‐D1‐pGADT7 with Cglac8‐pGBKT7 and MiLRR‐RLP1‐pGADT7 with Mi14‐3‐3‐D1‐pGBKT7 in yeast cells, screened on SD/−Trp−Leu−Ade−His medium. For the BiFC assay, co‐expression of MiLRR‐RLP1‐cYFP with Cglac8‐nYFP, Mi14‐3‐3‐D1‐cYFP with Cglac8‐nYFP and MiLRR‐RLP1‐cYFP with Mi14‐3‐3‐D1‐nYFP in *Nicotiana benthamiana* leaves. Subsequently, the leaves were stained with DAPI and used to observe the fluorescence signal 3 days after infiltration. The white arrow indicates the nucleus. Bars = 100 μm. For the pull‐down assay, the equivalent target proteins were co‐incubated with BeyoMag Protein A + G magnetic beads and anti‐Myc. Immunoblotting was performed with anti‐FLAG and anti‐Myc antibodies. The molecular docking was preformed using the HDOCK online website.

To investigate the interaction mechanism between Cglac8 of *C. gloeosporioides* and the host mango, we screened the interacting proteins using yeast two hybrid (Y2H) technology and obtained 34 different candidate mango proteins (Table S1). Among these candidate proteins, leucine‐rich repeat (LRR) receptor‐like protein At1g35710‐like and 14‐3‐3 were chosen for further analysis due to the evidence from previous studies that these two proteins are central to the immune system of plants (Sun et al. 2022; Obsilova and Obsil 2022; Zhao et al. 2021). Initially, we performed domain analysis and phylogenetic tree analysis to identify the structural domains and subfamily of the LRR protein. The result showed that the LRR protein is secreted (Figure S5a,b) and clustered into a large branch with LRR‐RLPs from different species (Figure S5c); it was named as MiLRR‐RLP1. The 14‐3‐3 protein, identified as a member of the mango 14‐3‐3 family and which contains a typical conserved pfam00244 domain (Xia et al. 2022), was named as Mi14‐3‐3‐D1. The Mi14‐3‐3‐D1 protein has no signal peptide (Figure S5d,e) but could be distributed extracellularly (Figure S5f). Moreover, *MiLRR‐RLP1* and *Mi14‐3‐3‐D1* exhibited tissue‐specific expression levels in mango (Figure S5g,h). To confirm the interaction between Cglac8, MiLRR‐RLP1 and Mi14‐3‐3‐D1, we initially performed Y2H point‐to‐point validation, showing that Cglac8, MiLRR‐RLP1 and Mi14‐3‐3‐D1 could interact with each other in yeast (Figure 3a). Bimolecular fluorescence complementation (BiFC) assay further demonstrated the interaction between Cglac8, MiLRR‐RLP1 and Mi14‐3‐3‐D1 in vivo (Figure 3b). This is consistent with the localisation of Cglac8, MiLRR‐RLP1 and Mi14‐3‐3‐D1 (Figure S6 and Figure 2a). Furthermore, the proteins of Cglac8, MiLRR‐RLP1 and Mi14‐3‐3‐D1 were expressed (Figure S7) and used for pull‐down assay. The results showed that recombinant Cglac8‐Myc protein could bind to MiLRR‐RLP1‐FLAG protein and Mi14‐3‐3‐D1‐FLAG protein. Consistently, Mi14‐3‐3‐D1‐Myc protein could bind to MiLRR‐RLP1‐FLAG protein (Figure 3c and Figure S8), suggesting that Cglac8, MiLRR‐RLP1 and Mi14‐3‐3‐D1 could directly interact with each other in vitro. Further confirmation was obtained through molecular docking analysis in silico (Figure 3d). Collectively, these results demonstrate the interaction between Cglac8, MiLRR‐RLP1 and Mi14‐3‐3‐D1 in vitro and in vivo.

### 2.4 *MiLRR‐RLP1* and *Mi14‐3‐3‐D1* Mediate Plant Resistance to *C. gloeosporioides*

**FIGURE 4:**
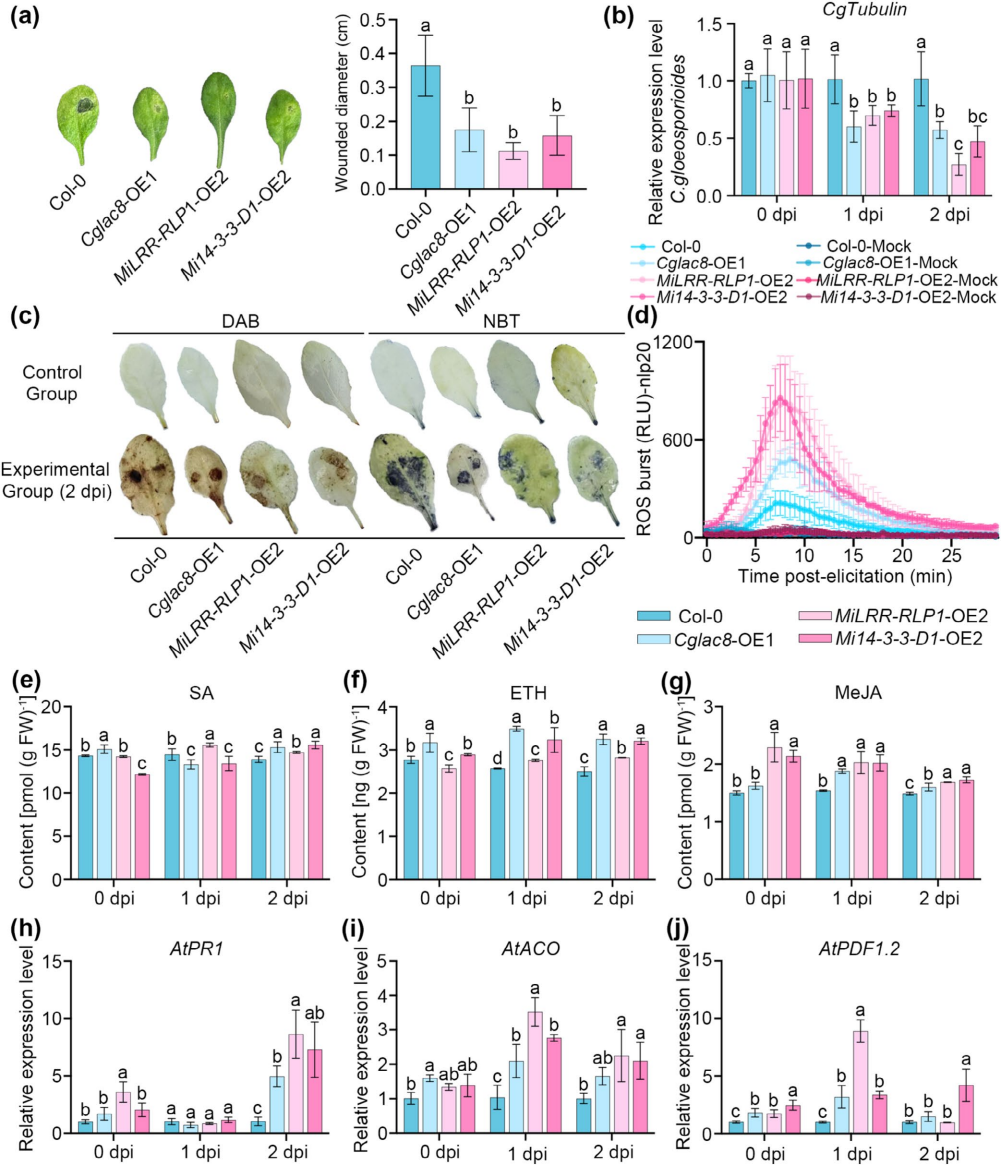
*MiLRR‐RLP1* and *Mi14‐3‐3‐D1* positively regulate resistance to *Colletotrichum gloeosporioides*. (a) The lesion diameter of *Arabidopsis* overexpression lines (OE) and wild‐type control (Col‐0). (b) The biomass of *C. gloeosporioides* in *Arabidopsis* leaves. The expression level of *CgTubulin* was used to quantify the accumulation of *C. gloeosporioides*. (c) 3,3'‐diaminobenzidine (DAB) and nitrotetrazolium blue (NBT) stainingf of *Arabidopsis* leaves. *Arabidopsis* leaves about 30 days old (Col‐0, *Cglac8*‐OE1, *MiLRR‐RLP1*‐OE2 and *Mi14‐3‐3‐D1*‐OE2) were inoculated with 2 × 10^6^ spores mL^−1^ conidial suspension of *C. gloeosporioides*, the phenotype was photographed and the diameter of the lesion was counted at 2 days post‐inoculation (dpi). (d) Dynamics of reactive oxygen species (ROS) accumulation induced by nlp20 peptide in Col‐0, *Cglac8*‐OE1, *MiLRR‐RLP1*‐OE2 and *Mi14‐3‐3‐D1*‐OE2 leaves. (e–g) The content of salicylic acid (SA) (e), ethylene (ETH) (f) and methyl jasmonate (MeJA) (g) in the leaves of Col‐0, *Cglac8*‐OE1, *MiLRR‐RLP1*‐OE2 and *Mi14‐3‐3‐D1*‐OE2 during *C. gloeosporioides* infection. (h–j) The expression levels of disease resistance‐related genes in the leaves of Col‐0, *Cglac8*‐OE1, *MiLRR‐RLP1*‐OE2 and *Mi14‐3‐3‐D1*‐OE2 during *C. gloeosporioides* infection. Different lowercase letters indicate significant differences at *p* < 0.05 (one‐way ANOVA).

To reveal the function of *Cglac8*, *MiLRR‐RLP1* and *Mi14‐3‐3‐D1* in response to *C. gloeosporioides* infection, the *Cglac8‐*OE1 (OE, overexpressed), *MiLRR‐RLP1‐*OE2 and *Mi14‐3‐3‐D1*‐OE2 *Arabidopsis* lines were generated (Figure S9). After 30 days of cultivation, *Cglac8*‐OE1 showed smaller leaf size and delayed reproductive growth; *MiLRR‐RLP1*‐OE2 exhibited significantly more rosette leaves; and *Mi14‐3‐3‐D1*‐OE2 showed relatively slower growth compared to the wild‐type Col‐0 (Figure S10). Compared with Col‐0, *Cglac8‐*OE1, *MiLRR‐RLP1‐*OE2 and *Mi14‐3‐3‐D1*‐OE2 significantly increased immune responses and disease resistance, including slighter symptoms, smaller lesion diameter and less *C. gloeosporioides* accumulation (Figure 4a,b). Moreover, Col‐0 leaves showed more intense 3,3'‐diaminobenzidine (DAB) and nitrotetrazolium blue chloride (NBT) staining than *Cglac8*‐OE1, *MiLRR‐RLP1*‐OE2 and *Mi14‐3‐3‐D1*‐OE2 leaves infected with *C. gloeosporioides* at 2 dpi (Figure 4c), indicating that Col‐0 accumulated more H_2_O_2_ and O^2−^. In addition, the uninoculated *Cglac8*‐OE1, *MiLRR‐RLP1*‐OE2 and *Mi14‐3‐3‐D1*‐OE2 leaves exhibited a significantly larger ROS burst than those in Col‐0 (Figure 4d). To further investigate the mechanism of *Cglac8, MiLRR‐RLP1* and *Mi14‐3‐3‐D1* in regulating host resistance to *C. gloeosporioides*, we measured the content of phytohormones (SA, ETH and MeJA) and transcripts of related genes in *Arabidopsis* leaves. The results revealed higher levels of both phytohormone content and related gene transcript levels in *Cglac8*‐OE1, *MiLRR‐RLP1*‐OE2 and *Mi14‐3‐3‐D1*‐OE2 leaves compared with the control (Figure 4e–j). These results indicate that *Cglac8*, *MiLRR‐RLP1* and *Mi14‐3‐3‐D1* are involved in the host infection response to *C. gloeosporioides*.

### 2.5 The Cglac8, MiLRR‐RLP1, Mi14‐3‐3‐D1 Combination Enhances Mango Immunity to *C. gloeosporioides*

**FIGURE 5:**
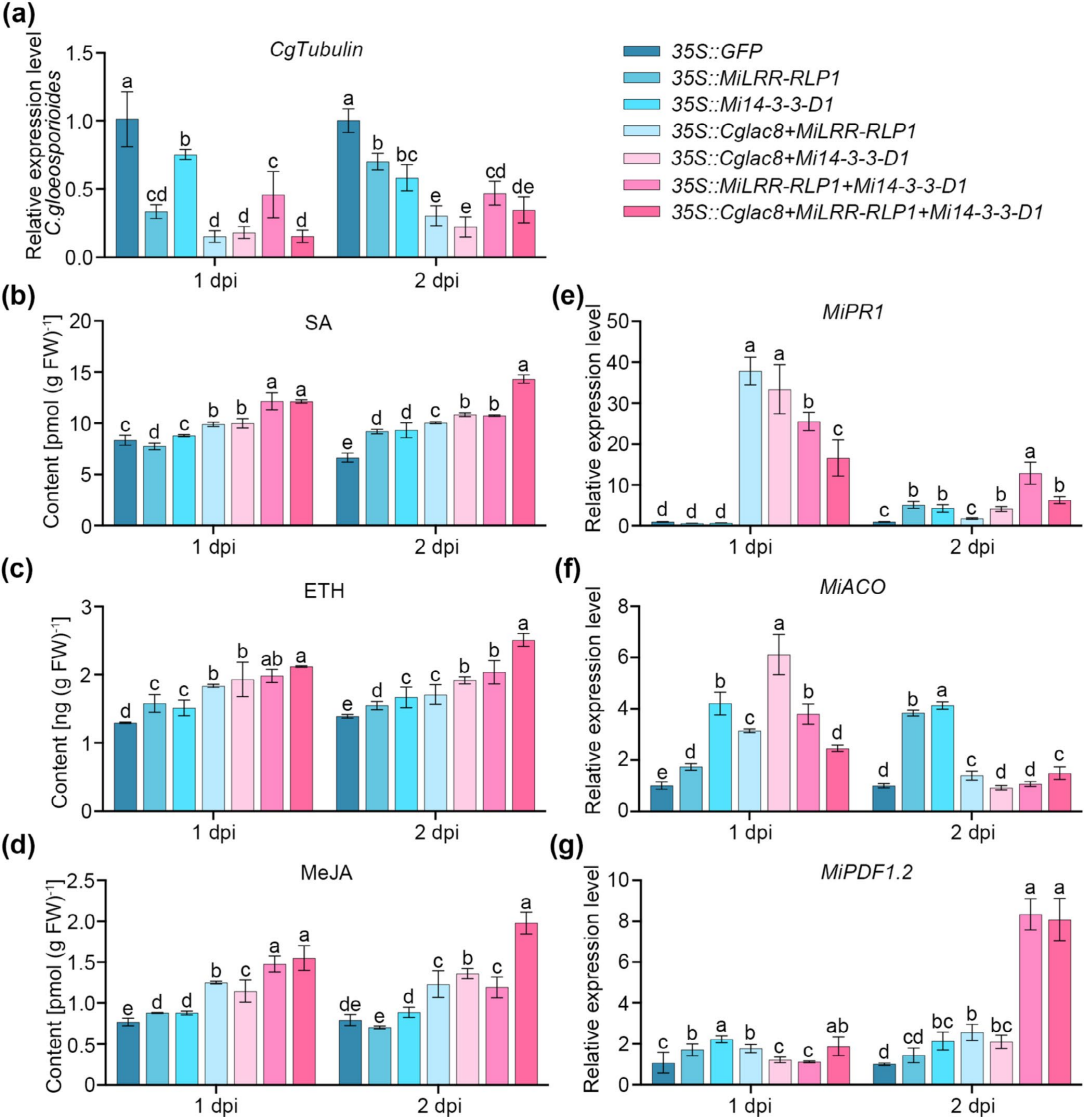
MiLRR‐RLP1 and Mi14‐3‐3‐D1 enhance Cglac8‐activated mango immunity. (a) The biomass of *Colletotrichum gloeosporioides* at 1 and 2 days post‐inoculation (dpi) (b–d) The level of salicylic acid (SA) (b), ethylene (ETH) (c) and methyl jasmonate (MeJA) (d) in mango leaves. (e–g) The expression level of *MiPR1* (e), *MiACO* (f) and *MiPDF1.2* (g) in overexpression mango leaves. The genes *GFP*, *MiLRR‐RLP1* and *Mi14‐3‐3‐D1* were transiently overexpressed in mango leaves singly;*Cglac8*, *MiLRR‐RLP1* and *Mi14‐3‐3‐D1* were expressed together in pairwise combinations and in three‐way combinations. Subsequently, the leaves were challenged with *C. gloeosporioides*. Then the leaves were collected and used for indicator measurement. Different lowercase letters indicate significant differences at *p* < 0.05 (one‐way ANOVA).

To verify whether *Cglac8* regulates *MiLRR‐RLP1*‐ and *Mi14‐3‐3‐D1*‐mediated immunity in mango, *35S::Cglac8 + MiLRR‐RLP1*, *35S::Cglac8 + Mi14‐3‐3‐D1*, *35S::MiLRR‐RLP1 + Mi14‐3‐3‐D1* and *35S::Cglac8 + MiLRR‐RLP1 + Mi14‐3‐3‐D1* were transiently expressed in mango leaves (Figure S4) and subsequently inoculated with *C. gloeosporioides* spore suspension. The results revealed that the biomass of *C. gloeosporioides* in overexpression leaves was significantly decreased (Figure 5a). Notably, the *35S::Cglac8 + MiLRR‐RLP1 + Mi14‐3‐3‐D1* leaves exhibited the lowest proportion of lesion area among those treatments at 5 dpi (Figure S11). Moreover, the contents of SA, ETH and MeJA exhibited higher levels in overexpression mango leaves than in the control leaves (Figure 5b–d). Interestingly, co‐overexpression combinations *35S::Cglac8 + MiLRR‐RLP1*, *35S::Cglac8 + Mi14‐3‐3‐D1*, *MiLRR‐RLP1 + Mi14‐3‐3‐D1* and *35S::Cglac8 + MiLRR‐RLP1 + Mi14‐3‐3‐D1* showed higher levels than the single overexpression *MiLRR‐RLP1* or *Mi14‐3‐3‐D1*. The expression level of *MiPR1* in *35S::Cglac8 + MiLRR‐RLP1*, *35S::Cglac8 + Mi14‐3‐3‐D1*, *35S::MiLRR‐RLP1 + Mi14‐3‐3‐D1* and *35S::Cglac8 + MiLRR‐RLP1 + Mi14‐3‐3‐D1* was significantly upregulated at 1 dpi, while decreasing at 2 dpi compared with 1 dpi, except for *35S::MiLRR‐RLP1* and *35S::Mi14‐3‐3‐D1* (Figure 5e). The expression level of *MiACO* was significantly upregulated in overexpression mango leaves at 1 dpi with the control (Figure 5f). The expression level of *MiPDF1.2* continued to increase, exhibiting differences between 1 dpi and 2 dpi (Figure 5g). Among them, the combination of *35S::MiLRR‐RLP1* + *Mi14‐3‐3‐D1* and *35S::Cglac8* + *MiLRR‐RLP1* + *Mi14‐3‐3‐D1* showed a more significant increase.

To analyse the function of MiLRR‐RLP1 and Mi14‐3‐3‐D1, we constructed mango plants silenced for MiLRR‐RLP1 or Mi14‐3‐3‐D1 by virus‐induced gene silencing (VIGS). Reverse transcription‐quantitative PCR (RT‐qPCR) confirmed that the transcriptional levels of *MiLRR‐RLP1* and *Mi14‐3‐3‐D1* were significantly downregulated in VIGS‐treated mango plants. The biomass of *C. gloeosporioides* that accumulated in VIGS‐silenced leaves significantly increased (Figure S12a). As shown in Figure S12b, the lesion area on the silenced leaves increased. Moreover, there were lower contents of SA, ETH and MeJA in silenced mango leaves compared to the control leaves (Figure S12c–e). Silencing also affected the transcriptional levels of *MiPR1*, *MiACO* and *MiPDF1.2* genes (Figure S12f–h).

Collectively, these results indicated that the expression of the binary complexes of *35S::Cglac8*, *35S::MiLRR‐RLP1* and *35S::Mi14‐3‐3‐D1*, as well as the co‐expression of these three proteins, can enhance the response level of mango to *C. gloeosporioides*. Moreover, MiLRR‐RLP1 and Mi14‐3‐3‐D1 enhanced the immune response of Cglac8‐activated mangoes to *C. gloeosporioides* infection.

## 3 Discussion

Controlling fungal diseases in tropical perennial fruit trees is a major challenge (Drenth and Guest 2016). Mango anthracnose is caused by the filamentous fungus *C. gloeosporioides* (Zhao et al. 2023), resulting in severe losses (Dofuor et al. 2023). However, plants have evolved multiple complex and effective innate immune mechanisms to cope with the biological stresses caused by pathogenic fungal infections (Atkinson and Urwin 2012). The apoplast is the first site of interaction, and also the most intense site of attack and defence between pathogenic fungi and hosts (Dora et al. 2022). Biotrophic and hemibiotrophic pathogens deliver effector proteins into the plant apoplast to modulate plant defences (Giraldo and Valent 2013; Shang et al. 2025). However, the interaction mechanisms between *C. gloeosporioides* and mango remain largely unknown. Therefore, further research is needed to investigate the mechanism of how mango can identify and monitor pathogens and activate immunity. Here, this study demonstrates that the mango receptor protein MiLRR‐RLP1 and protein Mi14‐3‐3‐D1 recognise the secreted Cglac8 of *C. gloeosporioides*, which can enhance plant immunity in response to *C. gloeosporioides* infection.

The formation of the penetration peg of *C. gloeosporioides* could cause plant cell wall distortion and increase intercellular spaces (Yu et al. 2022), providing a site for attack and defence interactions between *C. gloeosporioides* and hosts. Fungi can secrete laccase to destroy plant cell walls, oxidise phenolic and non‐phenolic substances, and promote their colonisation and growth (Liu et al. 2020). In this study, we identified the secreted laccase Cglac8 in *C. gloeosporioides*. *Cglac8* exhibited similar functions to *CgHOS2* (Liu et al. 2022), *CgGa1* (Li, Ke, et al. 2021) and *CgOPT2* (Li, Liu, et al. 2021), mainly regulation of conidia production and pathogenicity. Phytohormones are central regulators of plant defence, and their complex network of signalling pathways enables plants to activate appropriate defence responses against pathogens (Berens et al. 2017). Among them, SA is known for its regulatory role in the plant immune response, and activation of SA can trigger signalling pathways related to pathogen‐derived signal perception, defence gene expression, and induction of systemic acquired resistance (SAR) (Roychowdhury et al. 2024). Heterologous expression of Cglac8 in mango leaves could enhance the host's immunity to *C. gloeosporioides* infection via induced SA accumulation, *MiPR1* expression and ROS burst. MoORPs (from *Magnaporthe oryzae*) can induce disease resistance in *Arabidopsis* against *Pseudomonas syringae* pv. *tomato* DC3000 via stimulation of the ROS burst and *PR1* expression (Chen et al. 2022). We propose that Cglac8 could activate plant immunity through the induction of the accumulation of endogenous phytohormones such as SA, thereby resulting in the inhibition of *C. gloeosporioides* infection. The biological function of Cglac8 is both a virulence factor and an immune stimulator. This dual role may reflect a balance in host–pathogen co‐evolution: Cglac8 promotes infection through specific virulence‐related functions, such as cell wall degradation, while inadvertently activating immunity via host receptors. This interaction exemplifies a trade‐off inherent in the pathogenic strategy of *C. gloeosporioides*. Similarly, two effector proteins FocSIX1A and FocSIX6 from *Fusarium oxysporum* f. sp. *cubense*, overexpressed in xylem tissue of banana, can enhance resistance to vascular disease (Negi et al. 2025). In contrast, UgsL, a laccase from *Ustilaginoidea virens*, acts as a key role in regulating the virulence of the rice false smut fungus and enhances plant susceptibility to *U. virens* , *M. oryzae* and *Xanthomonas oryzae* pv. *oryzae* via inhibition of the PTI response (Duan et al. 2024). We propose that this may be related to the significant homology differences between UgsL and Cglac8 due to the wide biological functions of laccase encoded by plant‐pathogenic fungi.

**FIGURE 6:**
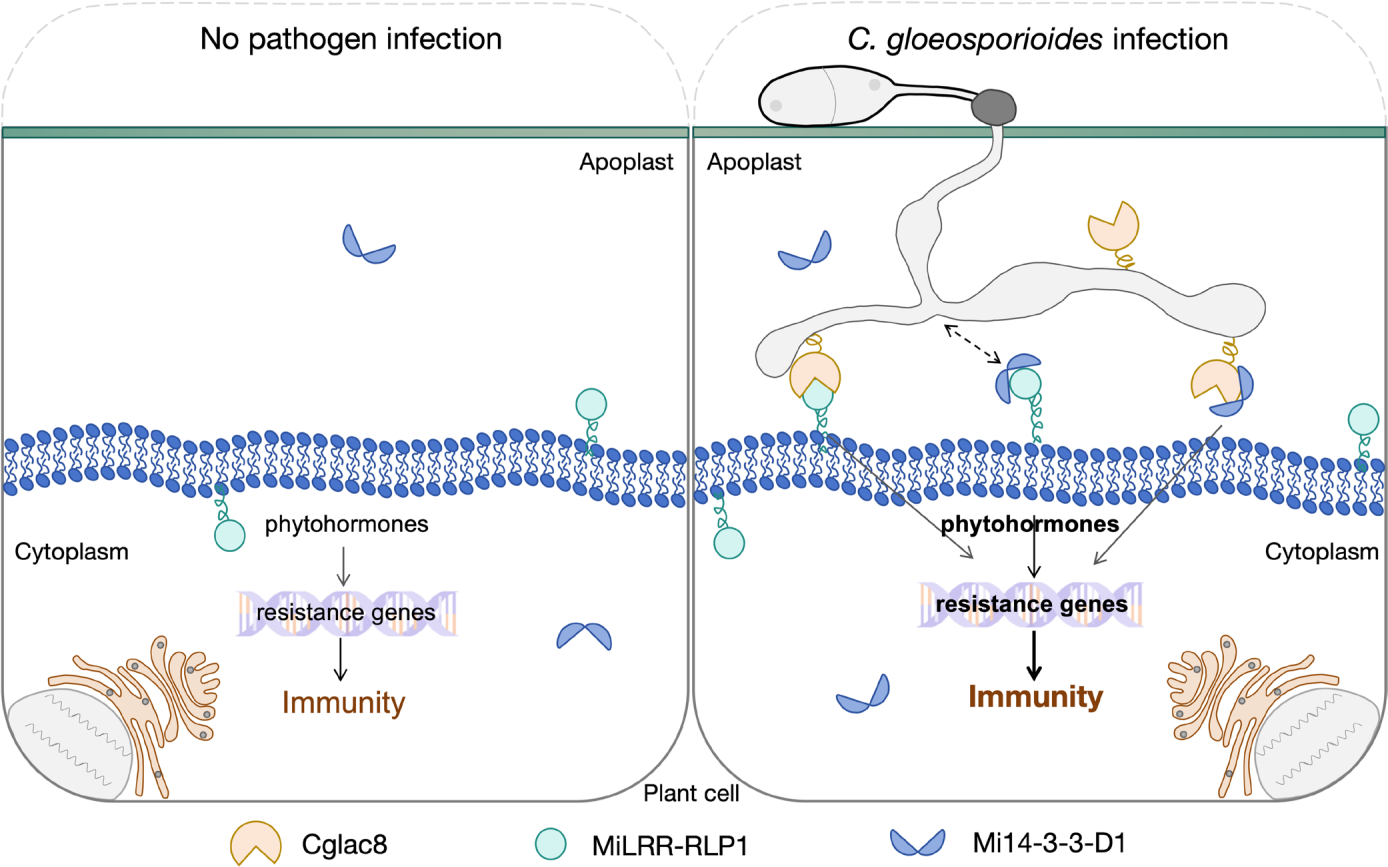
The proposed model of Cglac8 activation of MiLRR‐RLP1‐ and Mi14‐3‐3‐D1‐mediated plant immunity during *Colletotrichum gloeosporioides* infection.

Our research has established that there is an interaction between Cglac8, MiLRR‐RLP1 and Mi14‐3‐3‐D1. These proteins positively contribute to *C. gloeosporioides* resistance in mango by promoting the accumulation of ROS and phytohormones. *Arabidopsis* 14‐3‐3 proteins GRF6 and GRF8 positively regulate basal stomatal immunity against *P. syringae* pv. *tomato* DC3000 and *Botrytis cinerea* by mediating MAPKKK5 interaction and MAPKKK5 phosphorylation in *A. thaliana* (Dong et al. 2023). Previous studies have revealed that 14‐3‐3 protein can interact with NLR (Sheikh et al. 2023) and LRR‐RLKs (Rienties et al. 2005). In *Nicotiana*, the 14‐3‐3 protein acts as a scaffold between the N protein and helicase domains to support the interaction between R gene products and viral inducers (Konagaya et al. 2004). This current study confirmed that Cglac8, MiLRR‐RLP1 and Mi14‐3‐3‐D1 interact with each other in vitro and in vivo, and it was found that 14‐3‐3 can also interact with LRR‐RLP. Although we currently do not have direct evidence that these three proteins can form a stable complex, we speculate that Mi14‐3‐3‐D1 may regulate plant resistance to *C. gloeosporioides* by interacting with Cglac8 and MiLRR‐RLP1. Therefore, we overexpressed binary complexes of Cglac8, MiLRR‐RLP1 and Mi14‐3‐3‐D1, as well as an expression combination of all three proteins in mango leaves. After *C. gloeosporioides* infection, levels of phytohormone (for example, SA) were significantly higher in the overexpression combination Cglac8–MiLRR‐RLP1–Mi14‐3‐3‐D1 leaves than those in the control group, and the proportion of lesion area was significantly lower than that of the binary complex of Cglac8 with MiLRR‐RLP1 and Mi14‐3‐3‐D1. To compensate for the shortcomings of mango in silencing and transgenic systems, we also validated this experiment in tomato, the results of which were consistent with those in mango (Figures S13 and S14). In conclusion, our study indicated that MiLRR‐RLP1 and Mi14‐3‐3‐D1 could recognise and interact with Cglac8, and Mi14‐3‐3‐D1 may enhance plant resistance to *C. gloeosporioides* by participating in the interaction between Cglac8 and MiLRR‐RLP1 (Figure 6).

In this study, we confirmed that the plant immune response triggered by Cglac8 is dependent on MiLRR‐RLP1 and Mi14‐3‐3‐D1. Experiments have found that the co‐expression combination of secreted laccase Cglac8, plant disease resistance protein MiLRR‐RLP1 and Mi14‐3‐3‐D1 can enhance the plant response to *C. gloeosporioides* infection, with the overexpression of all three proteins together showing the most obvious response. Based on the characteristics of 14‐3‐3, we speculate that Mi14‐3‐3‐D1 may bind to the Cglac8‐MiLRR‐RLP1 module, thereby enhancing resistance to *C. gloeosporioides*. However, it remains unclear whether Mi14‐3‐3‐D1 functions as a scaffold protein and whether the complex formed is involved in changes in protein structure and laccase activity when it functions. How these three proteins form stable complexes and the role of Mi14‐3‐3‐D1 in this process remains to be confirmed.

## 4 Experimental Procedures

### 4.1 Materials and Growth Conditions

*Colletotrichum gloeosporioides* CATAS‐A2 was stored in test‐tube slants at 16°C in the laboratory (Zhang et al. 2023) and grown on potato dextrose agar (PDA) at 28°C in the dark. *M. indica* , *N. benthamiana*, *Solanum lycopersicoides* 'Micro‐Tom' and *A. thaliana* (Columbia‐0, Col‐0) were planted in a greenhouse at 25°C with 16 h light/8 h dark, relative humidity of 60%–70%. One‐year‐old grafted seedlings of mango with consistent growth were purchased from Danzhou Mango Germplasm Resources Garden of the Ministry of Agriculture and Rural Affairs. Then, they were transferred to indoor potted planting with high‐quality soil (soil:vermiculite:clay particles, 5:1:2). In addition, the fruit of *M. indica* (Tainung No. 1 mango), *Malus pumila* (Red Fuji apple) and *Pyrus bretschneideri* (Xuehua pear) were purchased from the store.

### 4.2 Treatment of Materials

The stem, leaves, flowers, fruits and seed tissues and organs of mango were collected from perennial plants, frozen in liquid nitrogen and stored at −80°C for further use. For the treatment of *C. gloeosporioides*, a 2 × 10^6^ spores mL^−1^ conidia suspension was sprayed uniformly on the newly formed tender shoot leaves of mango, then samples were collected at 0, 3, 6, 12, 24 and 48 h after inoculation and stored at −80°C for future use. For the genetic transformation of *A. thaliana* , transgenic overexpression lines *Cglac8*‐OE1, *MiLRR‐RLP1*‐OE2 and *Mi14‐3‐3‐D1*‐OE2 were obtained according to the pollen tube import method (Noman et al. 2019). Whole leaves and untreated leaves of *A. thaliana* were placed in 6‐well cell culture plates 3 days after being treated with *C. gloeosporioides*. The prepared NBT and DAB solutions were added (NBT solution configuration method refers to BCIP/NBT alkaline phosphatase colour development kit, DAB solution concentration is 1 mg mL^−1^). The NBT staining time was 6–8 h, and the DAB staining time was 24 h. After staining, the leaves were decolourised with ethanol and then photos were taken for observation.

### 4.3 RNA, DNA Extraction and RT‐qPCR

RNA was extracted using the TaKaRa MiniBEST Plant RNA Extraction Kit according to the instructions (TaKaRa). Then, cDNA was synthesised using the PrimeScript II 1st Strand cDNA Synthesis Kit (TaKaRa) with 1 μg of RNA. DNA was extracted using the CTAB method as described (Schenk et al. 2023). The real‐time qPCR was performed using SGExcel UltraSYBR Master premix (containing ROX) (Biotech) with internal reference genes of *MiActin* from *M. indica* , *SlActin* from *S. lycopersicoides*, *AtActin* from *A. thaliana* , and *CgTubulin* from *C. gloeosporioides*. qTOWER 2.0 (Analytik Jena) was used to analyse the fluorescence signal. Each data point was performed with at least three biological replicates. Primer sequences are listed in Table S2.

### 4.4 Construction of Plasmids, Transient Overexpression and VIGS in Plant Leaves

For the vector construction, the full‐length target genes were individually cloned into overexpression vector pUC57‐OE, secretion validation vector pSUC2, overexpression vector pEGAD, VIGS vector pTRV2, Y2H vector pGADT7/pGBKT7, BiFC vector pFGC‐nYFP/cYFP and prokaryotic expression vector pET‐32a (+), with the ClonExpress II One Step Cloning Kit (Vazyme) (Shi et al. 2024). All primers are listed in Table S2. For transient overexpression in plant leaves, *Agrobacterium tumefaciens* GV3101 carrying recombinant plasmids was used to treat the leaves (Lei et al. 2024; Pan et al. 2025), and subsequent experiments were conducted after 72 h of cultivation under low light conditions. For VIGS of *MiLRR‐RLP1* and *Mi14‐3‐3‐D1*, the main reference is Zhang et al. (2025).

### 4.5 Mutants Acquisition and Phenotype Determination

The correct knockout and complementation vectors of Cglac8 were used to obtain knockout mutant and complemented strain through PEG‐mediated protoplast transformation (Zhang et al. 2023) and CRISPR/Cas9‐mediated homology‐directed repair technology (Tan et al. 2025), respectively. The former is transferred into the wild‐type, while the latter is transferred into the knockout mutant and screened on the corresponding resistance plate. We observed colony growth and utilisation of phenolic substances of wild‐type, mutant ∆*Cglac8H* and complemented strain *C‐*∆*Cglac8H* on PDA, CM, CZA, PDA containing 30.0 g L^−1^ saccharose, 1 M potassium chloride, 1 M sodium chloride and 0.04% guaiacol, as well as pathogenicity determination on mangoes, apples and pears. For specific methods, refer to Zhang et al. (2023). The quantitative determination of fungal laccase was as described by Tan et al. (2025). The conidia of the strains were counted after shaking in potato glucose broth at 28°C and 180 rpm for 3 days. About 2 × 10^6^ spores mL^−1^ conidia suspension was used for maesuring conidial germination rate, at 0 h, 2 h, 4 h and 6 h with a DM2500 microscope (Leica). The penetration efficiency of appressoria was conducted at 6 h. Penetration efficiency (%) = (number of appressoria forming penetration pegs/total number of appressoria) × 100, with data from three biological replicates (100 appressoria counted per replicate).

The laccase assay mixture contained 0.5 mM 2, 2′‐azino‐bis (3‐ethylbenzothiazoline‐6‐sulfonic acid) (ABTS), 0.1 M sodium acetate (pH 4.5), a suitable amount of enzyme, and was incubated for 3 min at 45°C. Oxidation of ABTS was monitored by determining the increase in A_420_, with data from three biological replicates. The amount of laccase required for each milligram of protein to oxidise 1 nM of ABTS substrate per minute is one laccase enzyme activity unit.

### 4.6 Secretory Verification

Yeast invertase secretion assays were performed based on a previously described method (Meng et al. 2024). The recombinant plasmids pSUC2‐Cglac8 (the full‐length target gene with a secretory active signal peptide sequence) and pSUC2‐Cglac8^∆SP^ (the target gene without a signal peptide sequence) were individually transferred into the yeast strain YTK12. Then, the plate validation and TTC reduction colour reaction were performed as described by Chen et al. (2024). The plasmids pSUC2 and pSUC2‐PsAvr1b were used as negative and positive controls, respectively.

### 4.7 Bioinformatics Analysis

The structural domain and three‐dimensional structure of the target protein were predicted at SMART and SWISS‐MODEL online websites, respectively. The transmembrane domains and signal peptides were predicted at TMHMM‐2.0 and SignalP‐5.0 online websites, respectively. The multiple sequence alignment and phylogenetic tree were constructed via the neighbourhood‐joining method in MEGA 11 with seven leucine‐rich repeat receptor‐like proteins (LRR‐RLPs), seven leucine‐rich repeat receptor‐like kinases (LRR‐RLKs), and one chitin‐binding protein OsCEBiP (LysM) sequence downloaded from the NCBI database. The sequences are listed in Text S1.

### 4.8 Subcellular Localisation

The *A. tumefaciens* GV3101 carrying recombinant plasmids was injected into *N. benthamiana* leaves (Lei et al. 2024; Pan et al. 2025). After 3 days, the treated leaves were stained with DAPI solution for 1 h. For observing the separation of cytoplasmic walls, the leaves were also treated with 0.8 M NaCl solution. The fluorescence was observed using the laser confocal microscope (Olympus, Tokyo, Japan).

### 4.9 Y2H Assay

To confirm the interaction between Cglac8, MiLRR‐RLP1 and Mi14‐3‐3‐D1, the MiLRR‐RLP1‐pGADT7 and Cglac8‐pGBKT7, Mi14‐3‐3‐D1‐pGADT7 and Cglac8‐pGBKT7, and MiLRR‐RLP1‐pGADT7 and Mi14‐3‐3‐D1‐pGBKT7 recombinant plasmids were individually co‐transformed into yeast strain AH109 via PEG4000, and co‐transformed cells were plated on SD/−Trp−Leu and SD/−Trp−Leu−His−Ade media for 5 days at 28°C. Yeast cells co‐transformed with the empty vector were used as negative controls (Yan et al. 2021).

### 4.10 BiFC Assay

*Agrobacterium tumefaciens* GV3101 carrying MiLRR‐RLP1‐cYFP and Cglac8‐nYFP, Mi14‐3‐3‐D1‐cYFP and Cglac8‐nYFP and MiLRR‐RLP1‐cYFP & Mi14‐3‐3‐D1‐nYFP recombinant plasmid combinations were infiltrated into *N. benthamiana* leaves. After 3 days, the infiltrated leaves were stained with DAPI for 1 h, then the fluorescence was examined with the Olympus laser confocal microscope (Yan et al. 2021). The recombinant plasmid co‐transformed with empty vector were used as negative controls.

### 4.11 Pull‐Down Assay

The target proteins were induced and a pull‐down assay were performed as per Yan et al. (2021). Briefly, the proteins were induced overnight at 25°C with 1 mM isopropyl‐*β*‐D‐thiogalactopyranoside (IPTG). The protein combination was incubated at 4°C with BeyoMag Protein A + G magnetic beads (Beyotime) and anti‐Myc (Beyotime). After 10 h, the beads were washed with phosphate‐buffered saline (PBS) five times. Subsequently, the beads were boiled in a solution comprising 100 μL PBS and 25 μL SDS‐PAGE loading buffer for 10 min. The eluted proteins were detected by anti‐Myc and anti‐FLAG antibodies.

### 4.12 Molecular Docking Assay

For molecular docking technology, SWISS‐MODEL server was used to model the three‐dimensional structures of Cglac8, MiLRR‐RLP1 and Mi14‐3‐3‐D1. The interaction patterns and binding conformations between target proteins were analysed using the HDOCK online website. The results were analysed using PyMOL (v. 4.3.0) software to plot the amino acid residues interacting between the two proteins at a three‐dimensional angle.

### 4.13 Quantification of Phytohormone Content

To quantify the phytohormone levels, the leaves were weighed and ground at 4°C, then PBS was added and shaken to mix. The mixture was centrifuged at 8000 rpm for 10 min, then the supernatant was collected and used for quantification. According to the instructions, the levels of phytohormones (SA, ETH and MeJA) were quantified using the Plant SA ELISA Kit, Plant ETH ELISA Kit and Plant MeJA ELISA Kit (Enzyme Immunoassay).

### 4.14 Detection of ROS Burst

The ROS burst of Col‐0 and transgenic *Arabidopsis* overexpressiong lines was evaluated based on previously described methods (Yan et al. 2021). 1‐month‐old *Arabidopsis* leaf discs were collected and placed in a 96‐well plate with 50 μL sterile water for 12 h under dark conditions. Subsequently, sterile water was replaced with 100 μL of reaction solution containing 0.2 μM luminol, 10 μg mL^−1^ horseradish peroxidase and 20 μg mL^−1^ peptide nlp20 (VmNLP1) (Sangon Biotech) (Liu et al. 2021). The luminescence was immediately measured for 30 min using the LB960 microplate luminescence detector (Berthold). The luminescence reading is given in relative light units (RLU), which represents the content of ROS.

### 4.15 Data Analysis

All data were processed using IBM SPSS Statistics 26 and analysed using one‐way ANOVA, with lowercase letters indicating significant differences (*p* < 0.05).

## Author Contributions

M.Z.: Writing – original draft, data curation, methodology. J.P.: Methodology, formal analysis, investigation. C.L.: Validation. M.Z.: Software. A.G.: Conceptualization, funding acquisition. Y.C.: Resources, project administration. H.Z.: Writing – review and editing, supervision, visualisation.

## Conflicts of Interest

The authors declare no conflicts of interest.

## Supporting information


**Figure S1:** The expression level of *Cglac8* under *C. gloeosporioides* infection.

**Figure S2:** Structural analysis and signal peptide prediction of Cglac8. (a‐b) The sequence, structure and function of the laccase genes (a) and signal peptide (b) of Cglac8 were predicted via SMART (http://smart.embl‐heidelberg.de) and Signalp‐5.0 (https://services.healthtech.dtu.dk/services/SignalP‐5.0/), respectively.

**Figure S3:** Acquisition of *Cglac8* knockout and complementary mutants. (a) The flowchart for obtaining Cglac8 knockout and complementary mutants. (b) PCR validation of the knockout mutant ∆*Cglac8H* and complementary strain *C‐*∆*Cglac8H*. H852/H850 (F1/R1) was used to detect the insertion of *hygromycinB* (*hygB*) gene in knockout mutants. Cglac8H‐F/R (F2/R2) is a specific primer located in the upstream and downstream regions of Cglac8 DNA sequence. Bar‐F/R (F3/R3) was used to detect the glufosinate gene (*Bar*). Cglac8‐F/R (F4/R4) was used to detect the full length sequence of Cglac8. pUC57‐F/R (F5/R5) is the common primer of pUC57‐OE. pCglac8H, the plasmid used for knockout *Cglac8*, was used as positive control to detect the knockout of *Cglac8*. pC‐*∆*Cglac8H, the plasmid used for complementation *Cglac8*, was used as positive control to detect the complementary strain of *Cglac8*.

**Figure S4:** Transient overexpression of Cglac8, MiLRR‐RLP1 and Mi14‐3‐3‐D1 in mango. After treatment 2 days, the leaves were collected and used to extract RNA for further expression level analysis. A + B, A + C, B + C, A + B + C represent binary complexs, and co‐overexpressed with 3*5S::Cglac8*, 3*5S::MiLRR‐RLP1* and *35S::Mi14‐3‐3‐D1* three proteins combination. q*Cglac8*, q*MiLRR‐RLP1* and q*Mi14‐3‐3‐D1* were used as semiquantitative PCR primers for detecting Cglac8, MiLRR‐RLP1 and Mi14‐3‐3‐D1, while *MiActin* was used as semiquantitative PCR primers for detecting reference genes. lane 1–3: three repeats of the target gene; lane 4: empty vector (EV). Primer sequences were listed in Table S2.

**Figure S5:** The characteristic information of MiLRR‐RLP1 and Mi14‐3‐3‐D1. (a) The structural domain of MiLRR‐RLP1. (b) Verification of MiLRR‐RLP1 secretory. (c) The phylogenetic tree of MiLRR‐RLP1. The unrooted phylogenetic tree was constructed via MEGA 11 software by the neighbour‐joining method. The black circle represents MiLRR‐RLP1. (d) The structural domain of Mi14‐3‐3‐D1. (e) The signal peptide of Mi14‐3‐3‐D1. (f) The transmembrane domain analysis of Mi14‐3‐3‐D1. (g‐h) The expression level of *MiLRR‐RLP1* (g) and *Mi14‐3‐3‐D1* (h) in different tissues. The transcript level at seed was set as “1”.

**Figure S6:** Subcellular localization of MiLRR‐RLP1 and Mi14‐3‐3‐D1. (a‐b) The subcellular localization of MiLRR‐RLP1 (a) and Mi14‐3‐3‐D1 (b) in tobacco. The *Agrobacterium* GV3101 harbouring empty vector or the recombinant plasmids was infiltrated into *Nicotiana benthamiana* leaves. After 3 d, the fluorescence signal and DAPI‐stained cell nuclei in the infiltrated area were detected via a confocal laser‐scanning microscope. The white arrow indicates the nucleus. The dashed box shows the apoplast space formed after plasmolysis. Bars = 100 μm.

**Figure S7:** The prokaryotic expression of Cglac8, MiLRR‐RLP1 and Mi14‐3‐3‐D1.

**Figure S8:** The original images of Pull‐down experiment. The images show the chemiluminescence, bright field, and merged of Cglac8, MiLRR‐RLP1 and Mi14‐3‐3‐D1 interacting with each other. Number ①‐⑩ were the samples combinations involved in the experiment.

**Figure S9:** PCR verification of Arabidopsis transgenic lines. PCR validation of transgenic Arabidopsis *Cglac8, MiLRR‐RLP1* and *Mi14‐3‐3‐D1* overexpressing (OE) strain with primers Cglac8‐F/R, MiLRR‐RLP1‐F/R and Mi14‐3‐3‐D1‐F/R.

**Figure S10:** Morphology of whole plants and rosette leaves of Arabidopsis overexpression lines. The phenotype of wild type (Col‐0), *Cglac8*‐OE1, *MiLRR‐RLP1*‐OE2 and *Mi14‐3‐3‐D1*‐OE2 lines were observated after cultured 30 days. Bar = 1 cm.

**Figure S11:** MiLRR‐RLP1 & Mi14‐3‐3‐D1 enhance Cglac8 activated mango resistance to *C. gloeosporioides*. (a‐b) The phenotype (a) and lesion area (b) of mango leaves after infected with *C. gloeosporioides*. After constructed *35S::Cglac8*, *35S::Cglac8 + MiLRR‐RLP1*, *35S::Cglac8 + Mi14‐3‐3‐D1* and *35S::Cglac8 + MiLRR‐RLP1 + Mi14‐3‐3‐D1* plants, the *C. gloeosporioides* was individually inoculated in those leaves. After 5 d, the symptoms and lesion area detected. Different lowercase letters indicate significant differences at *p* < 0.05 (one‐way ANOVA).

**Figure S12:** Virus‐induced gene silencing of MiLRR‐RLP1, Mi14‐3‐3‐D1 genes. (a) The biomass of *C. gloeosporioides*. The phenotype (b) and the level of SA (c), ETH (d) and MeJA (e) at mango leaves. The expression level of *MiPR1* (f), *MiACO* (g) and *MiPDF1.2* (h) at virus‐induced gene silencing of MiLRR‐RLP1, Mi14‐3‐3‐D1 genes in mango leaves. Subsequently, infected them with *C. gloeosporioides*. Then the leaves were collected and used for indicator measurement. Different lowercase letters indicate significant differences at *p* < 0.05 (one‐way ANOVA).

**Figure S13:** The phytohormones content and expression levels of disease resistance related genes in tomato leaves after infection with *C. gloeosporioides*. (a) The biomass of *C. gloeosporioides* in co‐expressed tomato leaves after infection with *C. gloeosporioides*. (b‐d) Changes of SA (b), ETH (c) and MeJA (d) content in tomato leaves. (e‐g) The expression levels of *SlPR1* (e), *SlACO* (f) and *SlPI‐1* (g) in co‐expressed tomato leaves. After confirmed *GFP*, *MiLRR‐RLP1* and *Mi14‐3‐3‐D1* monomers, *35S::Cglac8*, *35S::MiLRR‐RLP1* and *35S::Mi14‐3‐3‐D1* pairwise combinations of binary complexes, and co‐overexpressed with three proteins combination were transient overexpression, the tomato leaves were infected with *C. gloeosporioides*. Different lowercase letters indicate significant differences at *p* < 0.05 (one‐way ANOVA).

**Figure S14:** Transient overexpression of Cglac8, MiLRR‐RLP1 and Mi14‐3‐3‐D1 in tomato. A + B, A + C, B + C, A + B + C represent binary complexs, and co‐overexpressed with *35S::Cglac8*, *35S::MiLRR‐RLP1* and *35S::Mi14‐3‐3‐D1* three proteins combination. q*Cglac8*, q*MiLRR‐RLP1* and q*Mi14‐3‐3‐D1* were used as semiquantitative PCR primers for detecting Cglac8, MiLRR‐RLP1 and Mi14‐3‐3‐D1, while *SlActin* was used as semiquantitative PCR primers for detecting reference genes. lane 1–3: three repeats of the target gene; lane 4: empty vector (EV).

**Table S1:** The candidate interaction proteins of Cglac8.

**Table S2:** The list of primers used in this study.

## Data Availability

The data that supports the findings of this study is available in the Supporting Information for this article. Supporting figures and tables related to the study can be found in Supporting Information Figures S1–, S14; Tables S1 and S2. The source data of the figures that support the findings of this study are listed in Database S1. For sequence information required for experimental integrity, refer to Text S1. Database S1 and Text S1 for Review but Not for Publication.
