# Supplementary figures and images for: A Secretory Protein Laccase lac8 From Pathogenic Fungi Activates Plant Protein 14‐3‐3 and Leucine‐Rich Repeat Receptor‐Like Protein LRR‐RLP1 to Trigger Mango Immunity
Source: Mol Plant Pathol. 2025 Oct 29;26(11):e70163. doi: 10.1111/mpp.70163 (PMC12571544; doi:10.1111/mpp.70163)

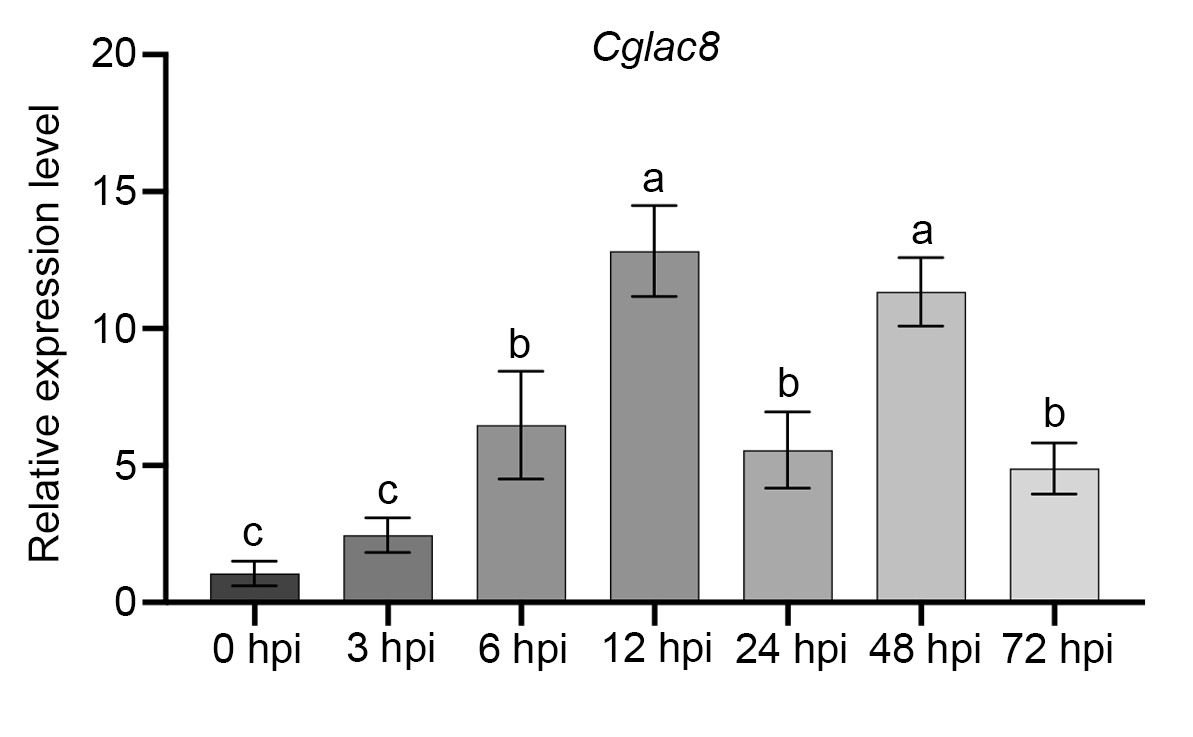

Supplement: Supplementary file 1 — Figure S1: The expression level of Cglac8 under C. gloeosporioides infection. [file MPP-26-e70163-s004.jpg]

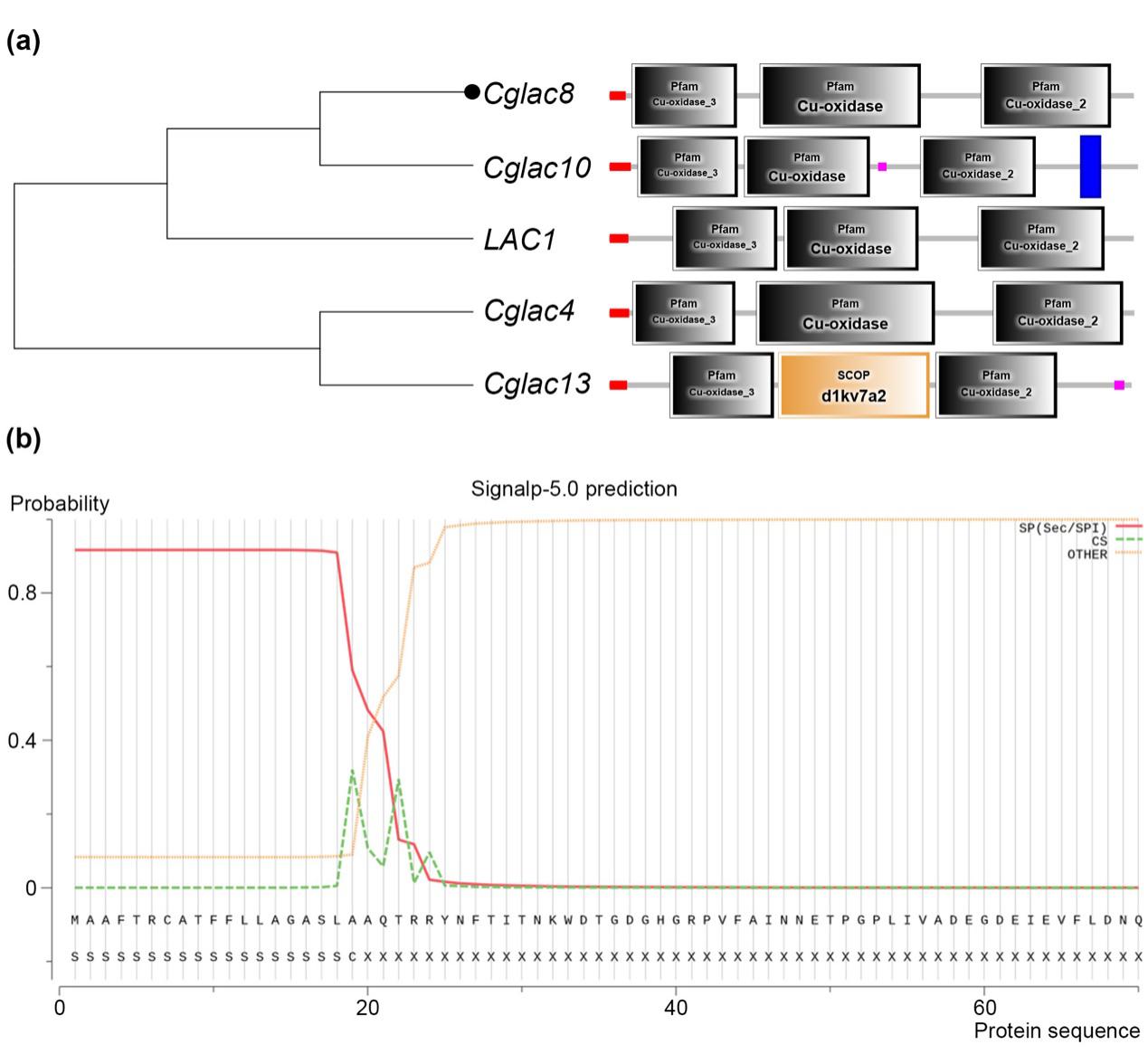

Supplement: Supplementary file 2 — Figure S2: Structural analysis and signal peptide prediction of Cglac8. (a‐b) The sequence, structure and function of the laccase genes (a) and signal peptide (b) of Cglac8 were predicted via SMART (http://smart.embl‐heidelberg.de) and Signalp‐5.0 (https://services.healthtech.dtu.dk/services/SignalP‐5.0/), respectively. [file MPP-26-e70163-s001.jpg]

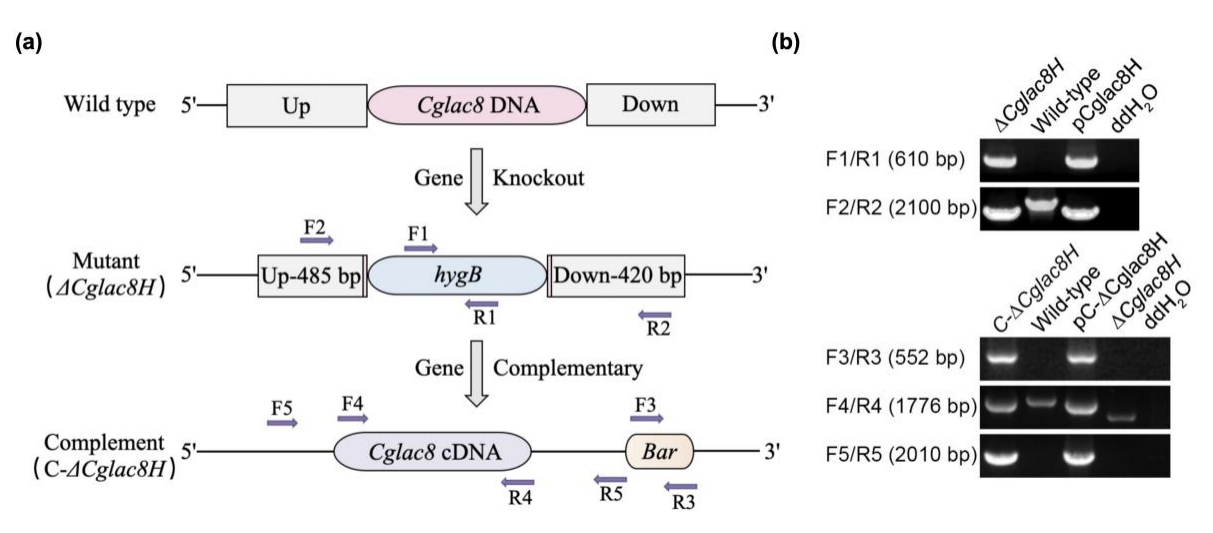

Supplement: Supplementary file 3 — Figure S3: Acquisition of Cglac8 knockout and complementary mutants. (a) The flowchart for obtaining Cglac8 knockout and complementary mutants. (b) PCR validation of the knockout mutant ∆Cglac8H and complementary strain C‐∆Cglac8H. H852/H850 (F1/R1) was used to detect the insertion of hygromycinB (hygB) gene in knockout mutants. Cglac8H‐F/R (F2/R2) is a specific primer located in the upstream and downstream regions of Cglac8 DNA sequence. Bar‐F/R (F3/R3) was used to detect the glufosinate gene (Bar). Cglac8‐F/R (F4/R4) was used to detect the full length sequence of Cglac8. pUC57‐F/R (F5/R5) is the common primer of pUC57‐OE. pCglac8H, the plasmid used for knockout Cglac8, was used as positive control to detect the knockout of Cglac8. pC‐∆Cglac8H, the plasmid used for complementation Cglac8, was used as positive control to detect the complementary strain of Cglac8. [file MPP-26-e70163-s007.jpg]

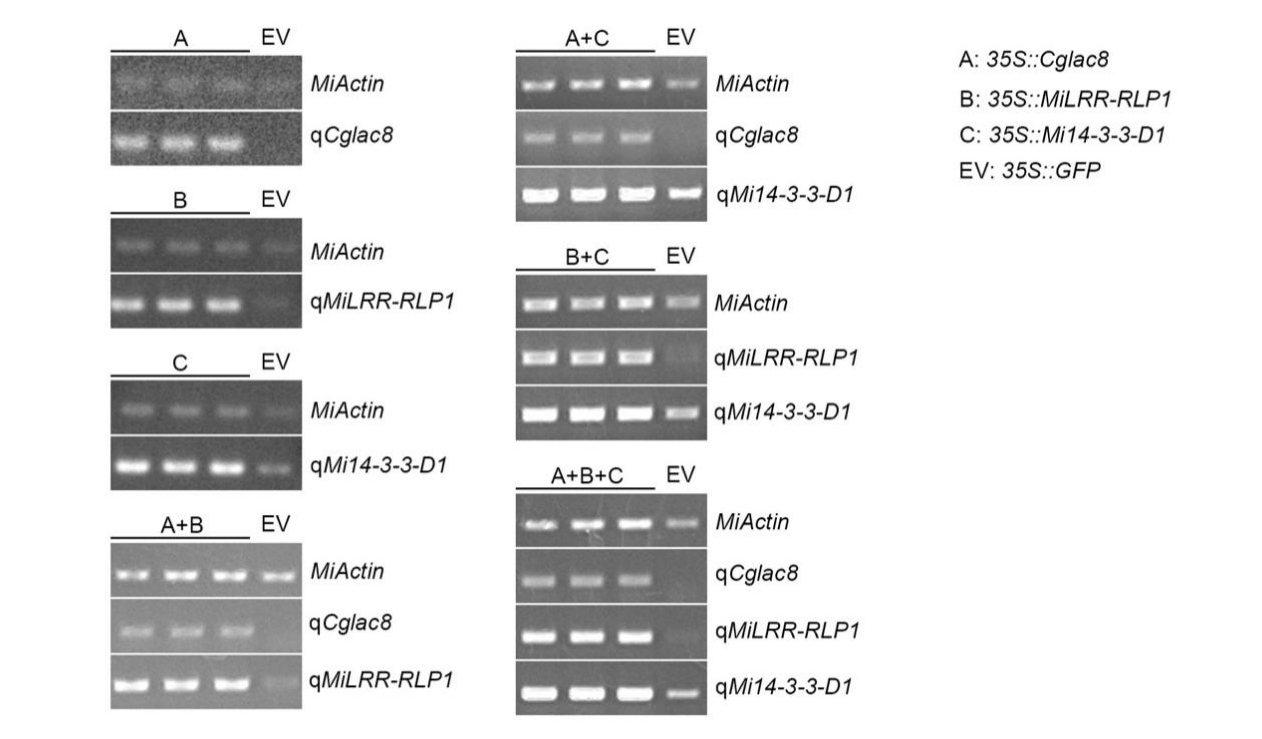

Supplement: Supplementary file 4 — Figure S4: Transient overexpression of Cglac8, MiLRR‐RLP1 and Mi14‐3‐3‐D1 in mango. After treatment 2 days, the leaves were collected and used to extract RNA for further expression level analysis. A + B, A + C, B + C, A + B + C represent binary complexs, and co‐overexpressed with 35S::Cglac8, 35S::MiLRR‐RLP1 and 35S::Mi14‐3‐3‐D1 three proteins combination. qCglac8, qMiLRR‐RLP1 and qMi14‐3‐3‐D1 were used as semiquantitative PCR primers for detecting Cglac8, MiLRR‐RLP1 and Mi14‐3‐3‐D1, while MiActin was used as semiquantitative PCR primers for detecting reference genes. lane 1–3: three repeats of the target gene; lane 4: empty vector (EV). Primer sequences were listed in Table S2. [file MPP-26-e70163-s009.jpg]

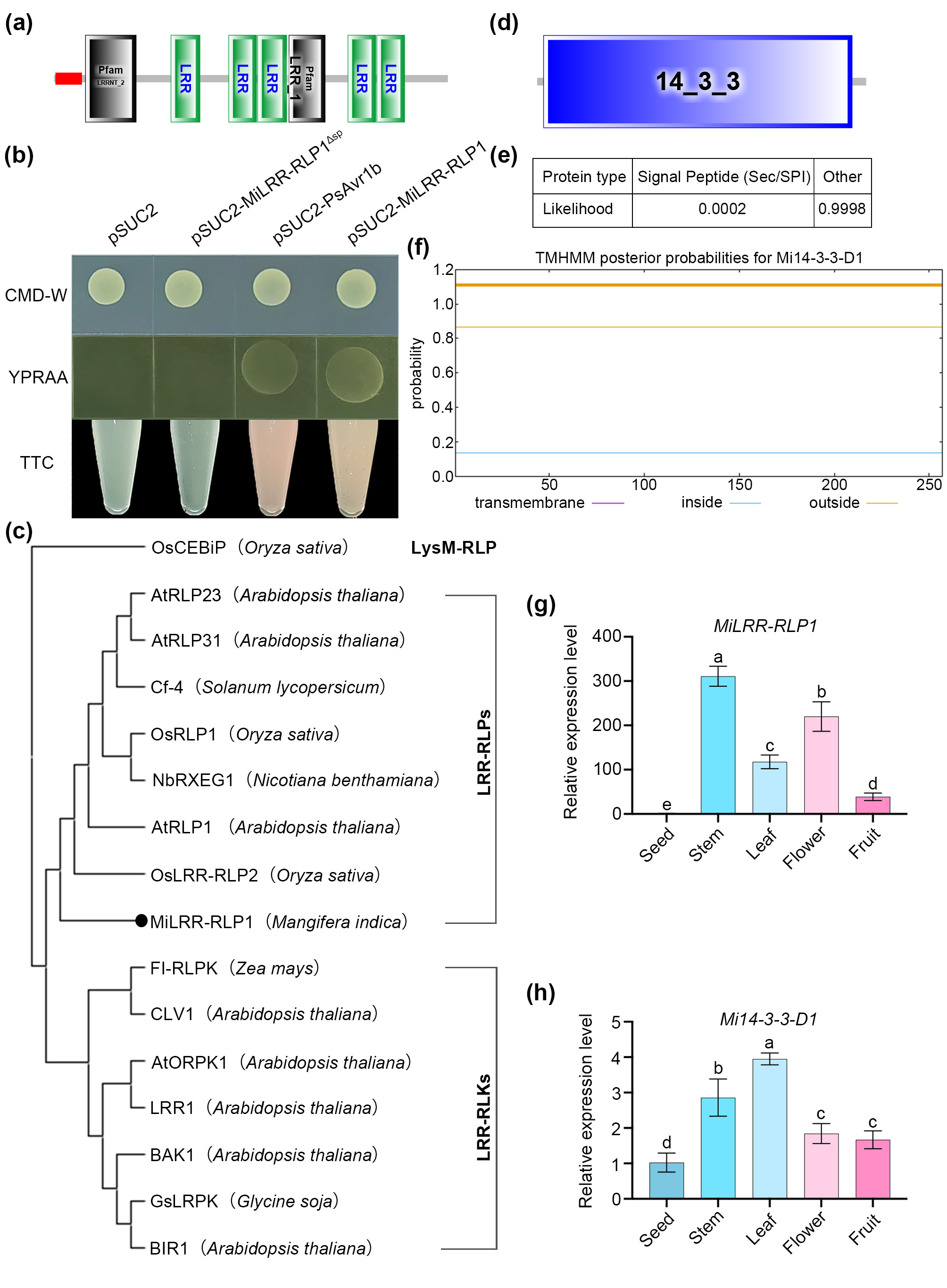

Supplement: Supplementary file 5 — Figure S5: The characteristic information of MiLRR‐RLP1 and Mi14‐3‐3‐D1. (a) The structural domain of MiLRR‐RLP1. (b) Verification of MiLRR‐RLP1 secretory. (c) The phylogenetic tree of MiLRR‐RLP1. The unrooted phylogenetic tree was constructed via MEGA 11 software by the neighbour‐joining method. The black circle represents MiLRR‐RLP1. (d) The structural domain of Mi14‐3‐3‐D1. (e) The signal peptide of Mi14‐3‐3‐D1. (f) The transmembrane domain analysis of Mi14‐3‐3‐D1. (g‐h) The expression level of MiLRR‐RLP1 (g) and Mi14‐3‐3‐D1 (h) in different tissues. The transcript level at seed was set as “1”. [file MPP-26-e70163-s011.jpg]

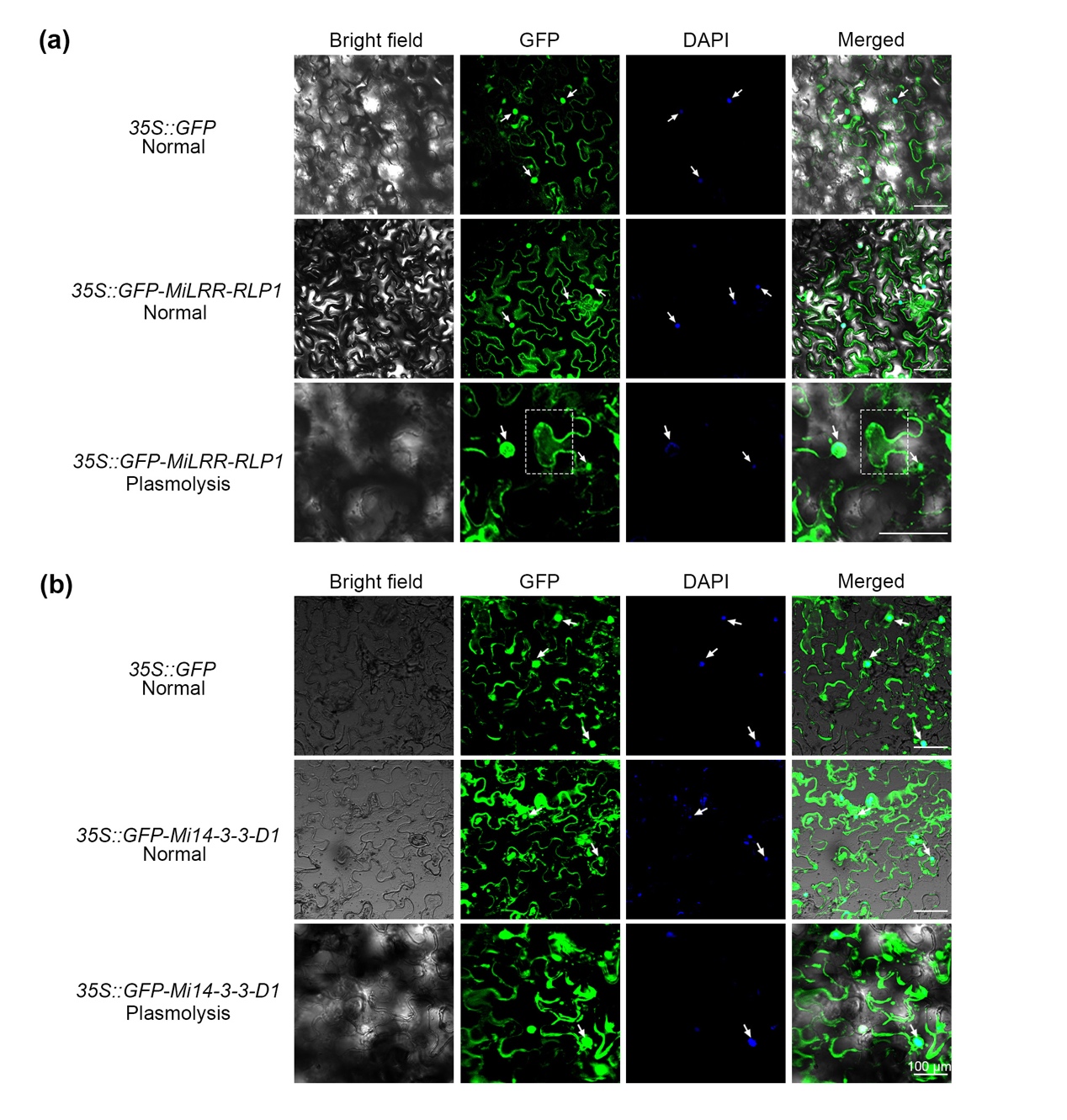

Supplement: Supplementary file 6 — Figure S6: Subcellular localization of MiLRR‐RLP1 and Mi14‐3‐3‐D1. (a‐b) The subcellular localization of MiLRR‐RLP1 (a) and Mi14‐3‐3‐D1 (b) in tobacco. The Agrobacterium GV3101 harbouring empty vector or the recombinant plasmids was infiltrated into Nicotiana benthamiana leaves. After 3 d, the fluorescence signal and DAPI‐stained cell nuclei in the infiltrated area were detected via a confocal laser‐scanning microscope. The white arrow indicates the nucleus. The dashed box shows the apoplast space formed after plasmolysis. Bars = 100 μm. [file MPP-26-e70163-s012.jpg]

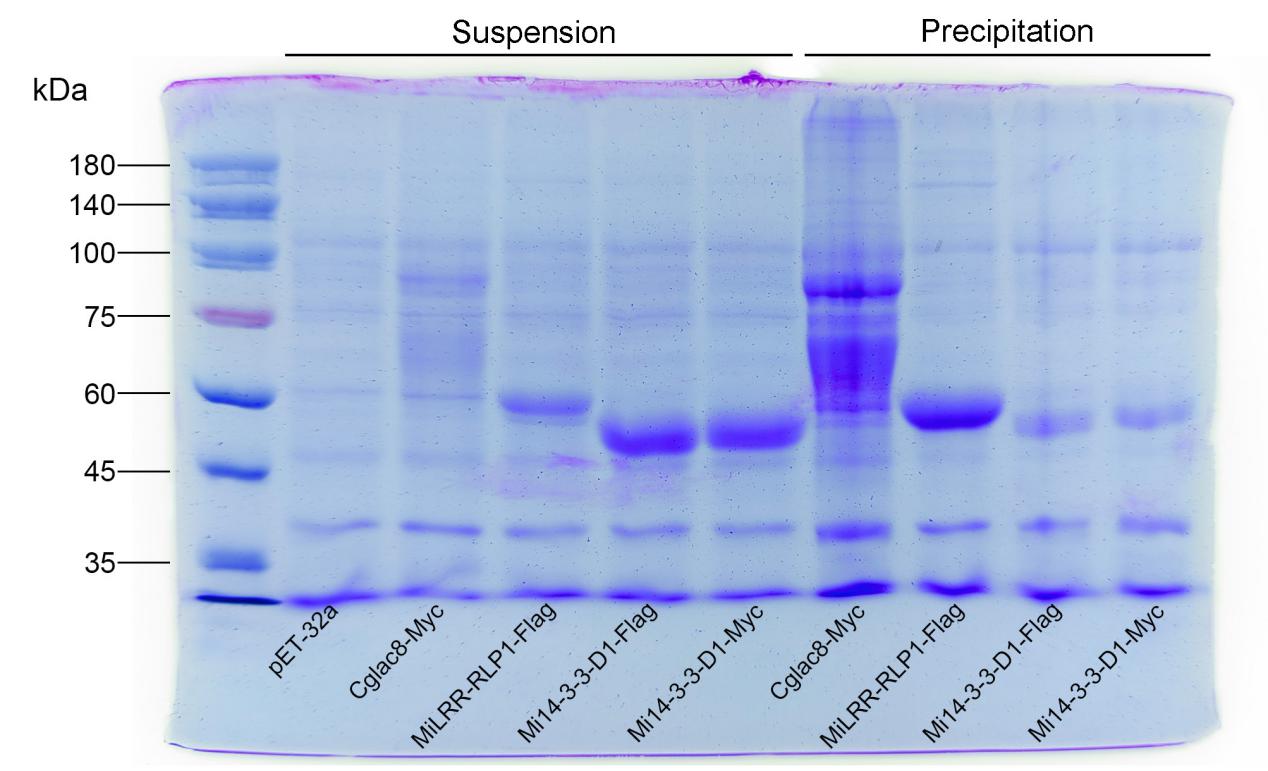

Supplement: Supplementary file 7 — Figure S7: The prokaryotic expression of Cglac8, MiLRR‐RLP1 and Mi14‐3‐3‐D1. [file MPP-26-e70163-s008.jpg]

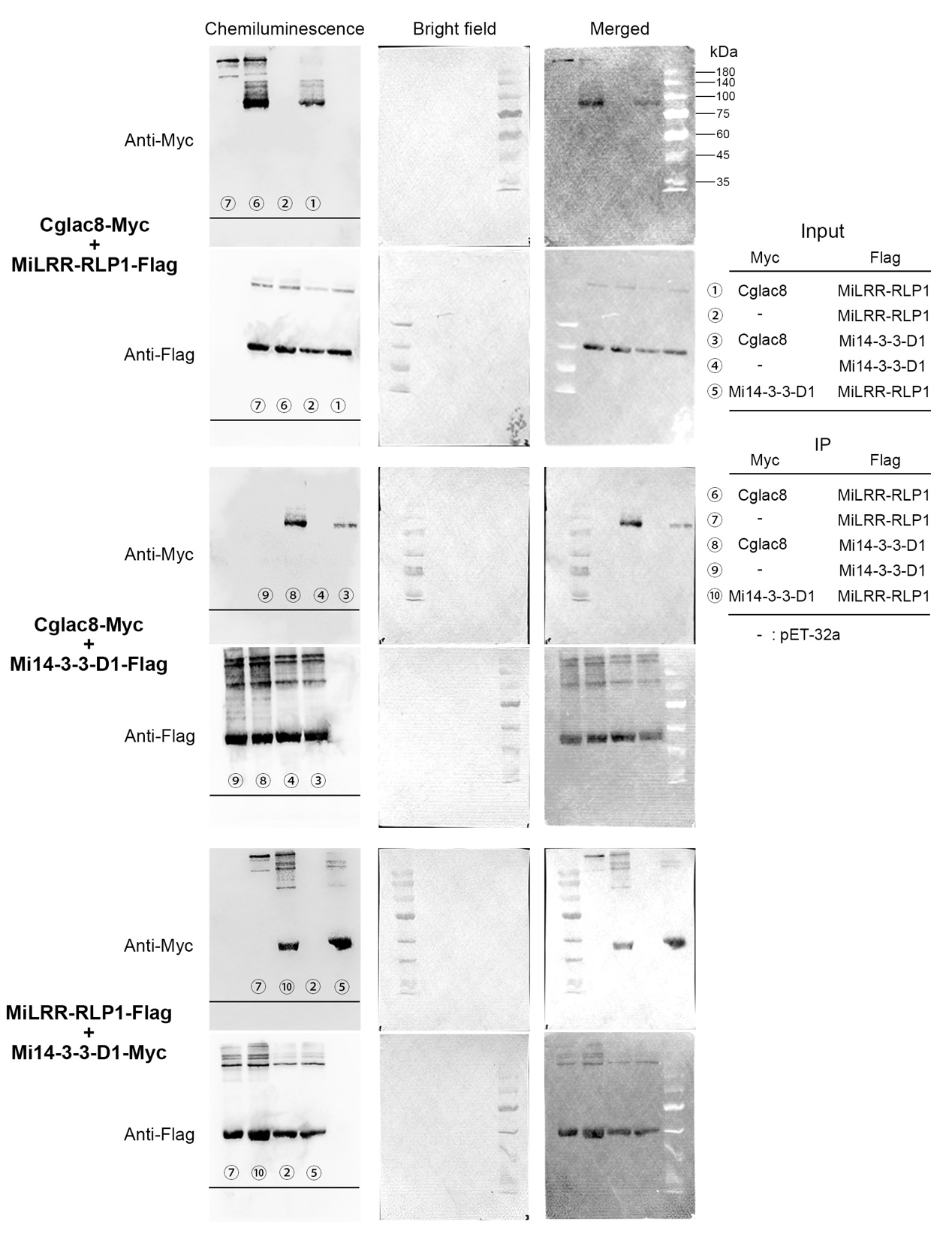

Supplement: Supplementary file 8 — Figure S8: The original images of Pull‐down experiment. The images show the chemiluminescence, bright field, and merged of Cglac8, MiLRR‐RLP1 and Mi14‐3‐3‐D1 interacting with each other. Number ①‐⑩ were the samples combinations involved in the experiment. [file MPP-26-e70163-s016.jpg]

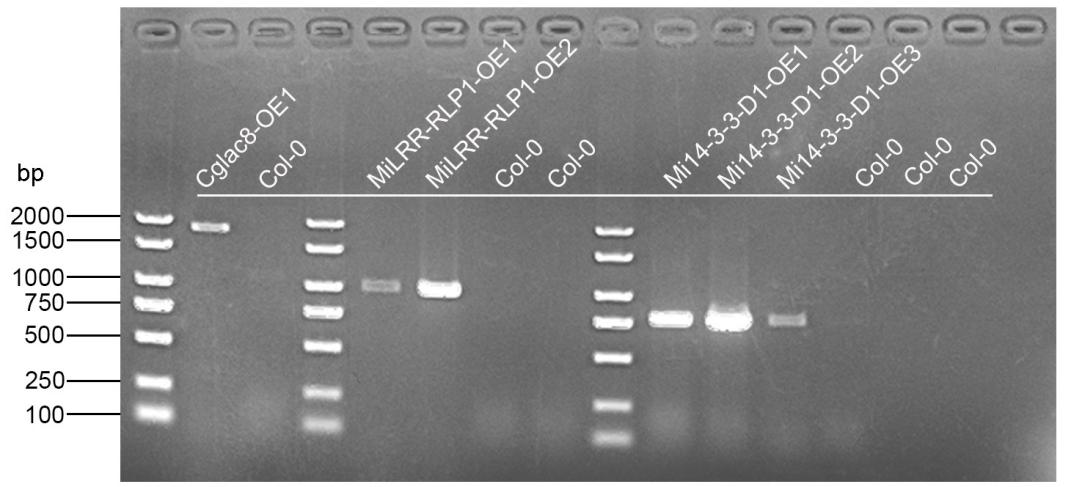

Supplement: Supplementary file 9 — Figure S9: PCR verification of Arabidopsis transgenic lines. PCR validation of transgenic Arabidopsis Cglac8, MiLRR‐RLP1 and Mi14‐3‐3‐D1 overexpressing (OE) strain with primers Cglac8‐F/R, MiLRR‐RLP1‐F/R and Mi14‐3‐3‐D1‐F/R. [file MPP-26-e70163-s005.jpg]

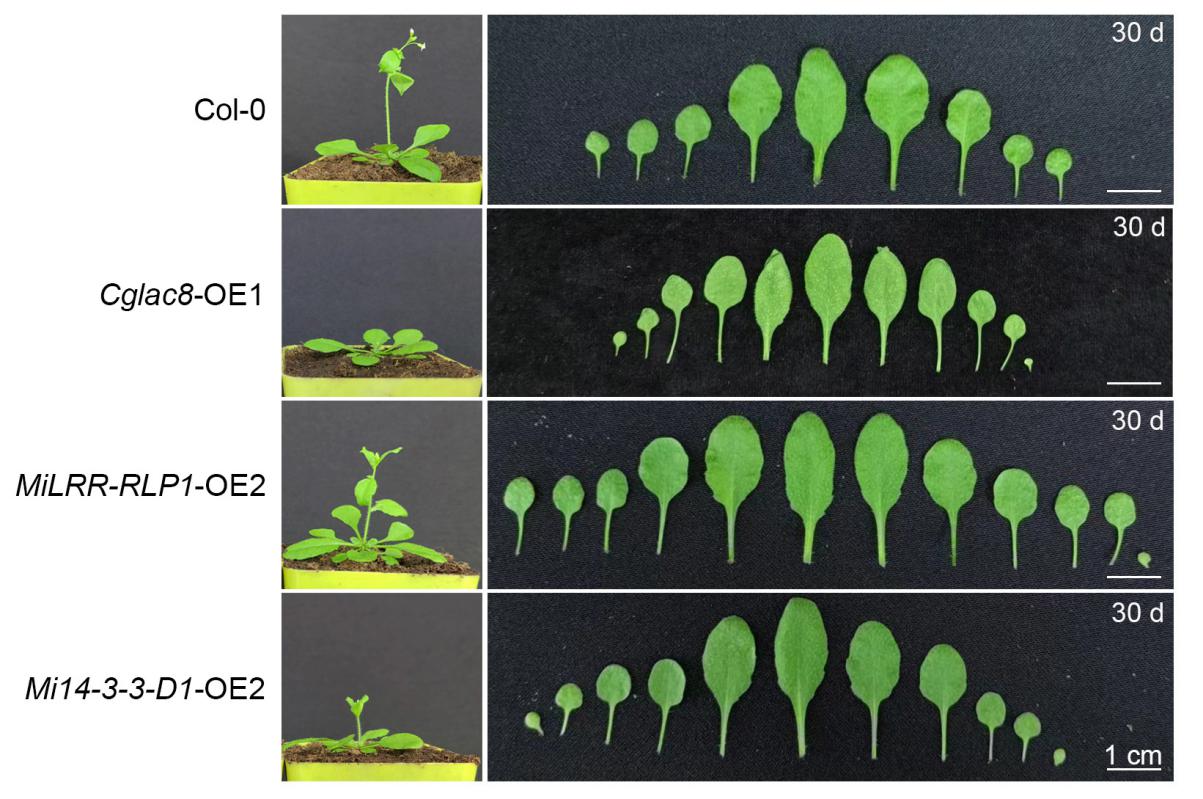

Supplement: Supplementary file 10 — Figure S10: Morphology of whole plants and rosette leaves of Arabidopsis overexpression lines. The phenotype of wild type (Col‐0), Cglac8‐OE1, MiLRR‐RLP1‐OE2 and Mi14‐3‐3‐D1‐OE2 lines were observated after cultured 30 days. Bar = 1 cm. [file MPP-26-e70163-s010.jpg]

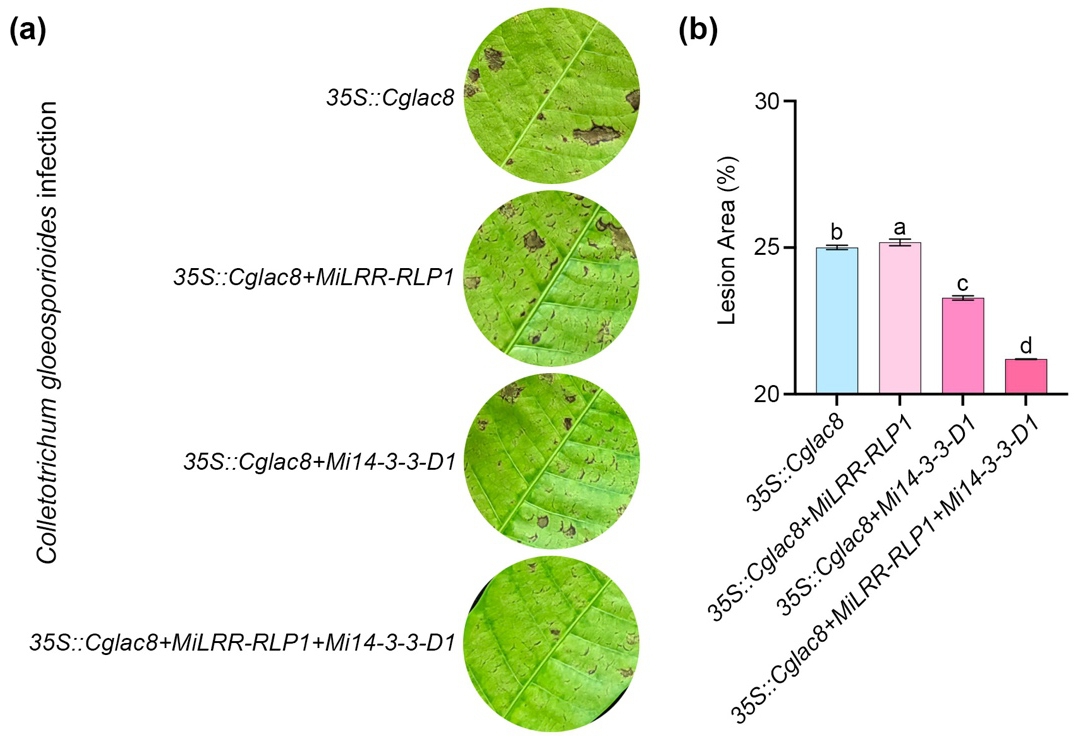

Supplement: Supplementary file 11 — Figure S11: MiLRR‐RLP1 & Mi14‐3‐3‐D1 enhance Cglac8 activated mango resistance to C. gloeosporioides. (a‐b) The phenotype (a) and lesion area (b) of mango leaves after infected with C. gloeosporioides. After constructed 35S::Cglac8, 35S::Cglac8 + MiLRR‐RLP1, 35S::Cglac8 + Mi14‐3‐3‐D1 and 35S::Cglac8 + MiLRR‐RLP1 + Mi14‐3‐3‐D1 plants, the C. gloeosporioides was individually inoculated in those leaves. After 5 d, the symptoms and lesion area detected. Different lowercase letters indicate significant differences at p < 0.05 (one‐way ANOVA). [file MPP-26-e70163-s013.jpg]

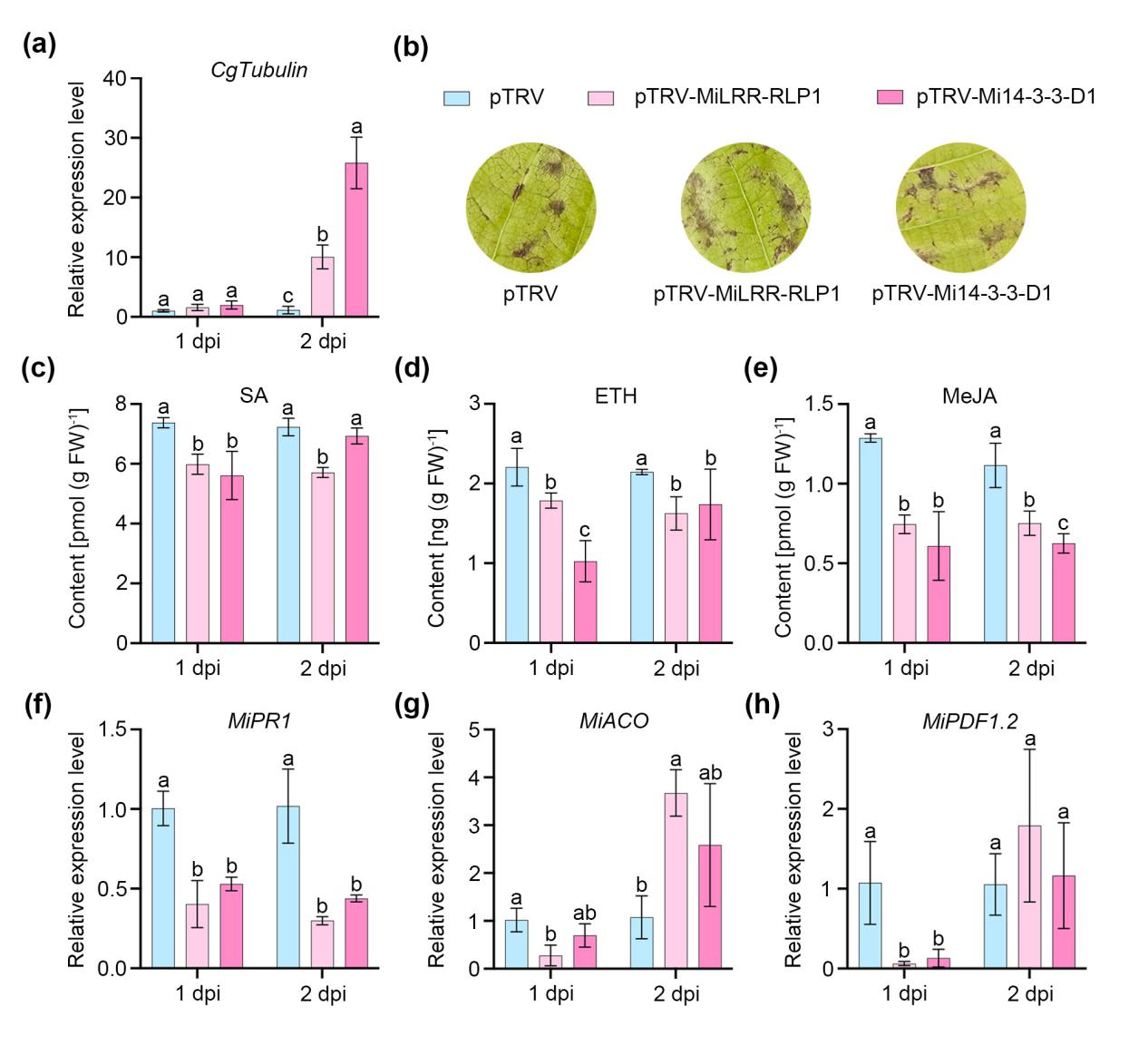

Supplement: Supplementary file 12 — Figure S12: Virus‐induced gene silencing of MiLRR‐RLP1, Mi14‐3‐3‐D1 genes. (a) The biomass of C. gloeosporioides. The phenotype (b) and the level of SA (c), ETH (d) and MeJA (e) at mango leaves. The expression level of MiPR1 (f), MiACO (g) and MiPDF1.2 (h) at virus‐induced gene silencing of MiLRR‐RLP1, Mi14‐3‐3‐D1 genes in mango leaves. Subsequently, infected them with C. gloeosporioides. Then the leaves were collected and used for indicator measurement. Different lowercase letters indicate significant differences at p < 0.05 (one‐way ANOVA). [file MPP-26-e70163-s006.jpg]

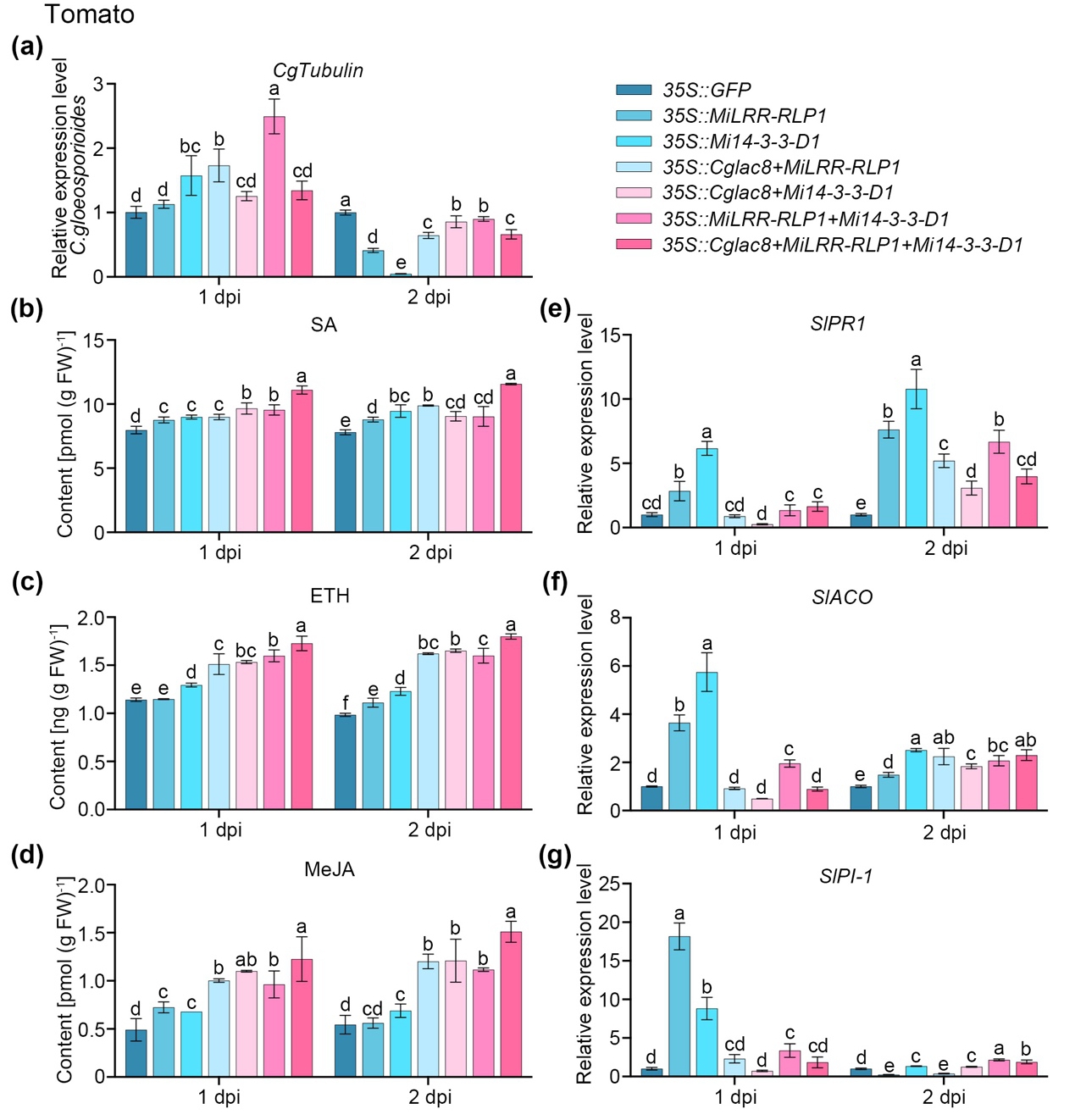

Supplement: Supplementary file 13 — Figure S13: The phytohormones content and expression levels of disease resistance related genes in tomato leaves after infection with C. gloeosporioides. (a) The biomass of C. gloeosporioides in co‐expressed tomato leaves after infection with C. gloeosporioides. (b‐d) Changes of SA (b), ETH (c) and MeJA (d) content in tomato leaves. (e‐g) The expression levels of SlPR1 (e), SlACO (f) and SlPI‐1 (g) in co‐expressed tomato leaves. After confirmed GFP, MiLRR‐RLP1 and Mi14‐3‐3‐D1 monomers, 35S::Cglac8, 35S::MiLRR‐RLP1 and 35S::Mi14‐3‐3‐D1 pairwise combinations of binary complexes, and co‐overexpressed with three proteins combination were transient overexpression, the tomato leaves were infected with C. gloeosporioides. Different lowercase letters indicate significant differences at p < 0.05 (one‐way ANOVA). [file MPP-26-e70163-s003.jpg]

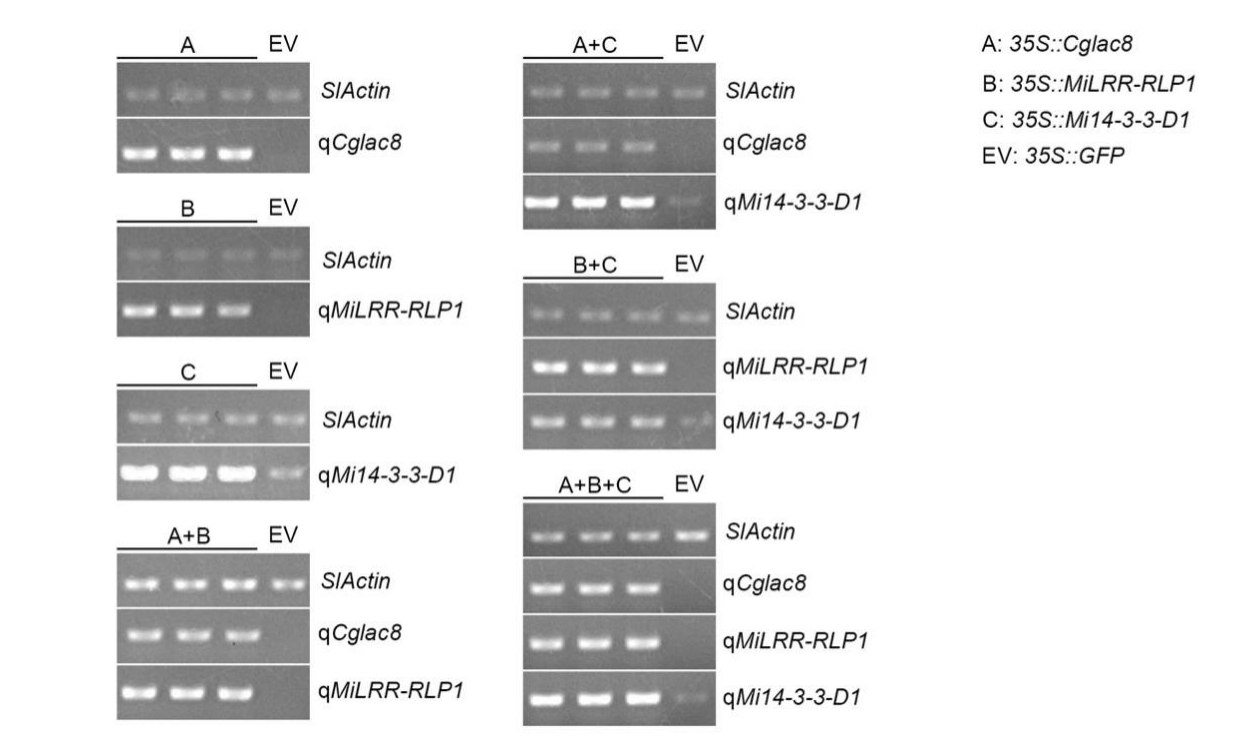

Supplement: Supplementary file 14 — Figure S14: Transient overexpression of Cglac8, MiLRR‐RLP1 and Mi14‐3‐3‐D1 in tomato. A + B, A + C, B + C, A + B + C represent binary complexs, and co‐overexpressed with 35S::Cglac8, 35S::MiLRR‐RLP1 and 35S::Mi14‐3‐3‐D1 three proteins combination. qCglac8, qMiLRR‐RLP1 and qMi14‐3‐3‐D1 were used as semiquantitative PCR primers for detecting Cglac8, MiLRR‐RLP1 and Mi14‐3‐3‐D1, while SlActin was used as semiquantitative PCR primers for detecting reference genes. lane 1–3: three repeats of the target gene; lane 4: empty vector (EV). [file MPP-26-e70163-s002.jpg]
