# Supplementary material for: A Secretory Protein Laccase lac8 From Pathogenic Fungi Activates Plant Protein 14‐3‐3 and Leucine‐Rich Repeat Receptor‐Like Protein LRR‐RLP1 to Trigger Mango Immunity
Source: Mol Plant Pathol. 2025 Oct 29;26(11):e70163. doi: 10.1111/mpp.70163 (PMC12571544; doi:10.1111/mpp.70163)
Supplement: Supplementary file 15 — Table S1: The candidate interaction proteins of Cglac8. [file MPP-26-e70163-s014.doc]

**Table S1. The candidate interaction proteins of Cglac8.**

| Number | Protein description | login number | protein (aa) |
| --- | --- | --- | --- |
| 1 | sphingoid long-chain bases kinase 1 | XP_044490157.1 | 777 |
| 2 | disease resistance protein At4g27190-like | XP_044477939.1 | 1162 |
| 3 | 40S ribosomal protein S8-like | XP_044502964.1 | 224 |
| **4** | **probable leucine-rich repeat receptor-like protein At1g35710** | XP_044488279.1 | 327 |
| 5 | protein PHLOEM PROTEIN 2-LIKE A1-like isoform X1 | XP_044496828.1 | 204 |
| 6 | protein ENHANCED DISEASE RESISTANCE 4-like isoform X1 | XP_044477224.1 | 948 |
| 7 | protein COFACTOR ASSEMBLY OF COMPLEX C SUBUNIT B CCB1, chloroplastic | XP_044502911.1 | 268 |
| 8 | nifU-like protein 2, chloroplastic | XP_044488000.1 | 232 |
| 9 | uncharacterized protein LOC123193128 | XP_044461847.1 | 203 |
| 10 | probable pyridoxal 5'-phosphate synthase subunit PDX2 | XP_044473105.1 | 267 |
| 11 | BURP domain-containing protein 3-like | XP_044464593.1 | 360 |
| 12 | protein SAMBA | XP_044466950.1 | 112 |
| 13 | thiamine thiazole synthase, chloroplastic | XP_044474138.1 | 354 |
| 14 | 60S ribosomal protein L31 | XP_044471592.1 | 120 |
| **15** | **14-3-3-D1 protein** | UVF71843.1 | 257 |
| 16 | ATP-dependent Clp protease proteolytic subunit-related protein 1, chloroplastic-like isoform X2 | XP_044507310.1 | 385 |
| 17 | phospholipase A1-II 4-like, partial | XP_044497618.1 | 237 |
| 18 | vacuolar protein sorting-associated protein 32 homolog 2-like | XP_044480074.1 | 218 |
| 19 | glutamine synthetase leaf isozyme, chloroplastic | XP_044484022.1 | 432 |
| 20 | uncharacterized protein LOC123221020 | XP_044499620.1 | 660 |
| 21 | chlorophyll a-b binding protein CP24 10A, chloroplastic | XP_044491153.1 | 257 |
| 22 | chloroplast chlorophyll A/B binding protein | ACR08639.1 | 264 |
| 23 | peptidyl-prolyl cis-trans isomerase-like | XP_044509353.1 | 172 |
| 24 | protein translation factor SUI1 homolog 2 isoform X1 | XP_044472657.1 | 113 |
| 25 | divinyl chlorophyllide a 8-vinyl-reductase, chloroplastic-like | XP_044500827.1 | 420 |
| 26 | uncharacterized protein LOC123224014 | XP_044503433.1 | 229 |
| 27 | chlorophyll a-b binding protein P4, chloroplastic-like | XP_044477011.1 | 247 |
| 28 | glycine dehydrogenase (decarboxylating), mitochondrial-like | XP_044463891.1 | 1054 |
| 29 | 60S ribosomal protein L18a-2 | XP_031280548.1 | 178 |
| 30 | 50S ribosomal protein L11, chloroplastic-like | XP_044484693.1 | 234 |
| 31 | UPF0664 stress-induced protein C29B12.11c-like | XP_044508691.1 | 204 |
| 32 | probable small nuclear ribonucleoprotein Sm D2 isoform X2 | XP_044491885.1 | 103 |
| 33 | F-box/kelch-repeat protein At5g42350-like | XP_044511643.1 | 581 |
| 34 | PHD finger protein EHD3-like isoform X4 | XP_044479291.1 | 506 |
