# Supplementary material for: A Secretory Protein Laccase lac8 From Pathogenic Fungi Activates Plant Protein 14‐3‐3 and Leucine‐Rich Repeat Receptor‐Like Protein LRR‐RLP1 to Trigger Mango Immunity
Source: Mol Plant Pathol. 2025 Oct 29;26(11):e70163. doi: 10.1111/mpp.70163 (PMC12571544; doi:10.1111/mpp.70163)
Supplement: Supplementary file 16 — Table S2: The list of primers used in this study. [file MPP-26-e70163-s015.doc]

**Table S2. The list of primers used in this study.**

|  | Primer | Sequence（5’-3’） |
| --- | --- | --- |
|  | Cglac8-F (F4) | ATGGCTGCCTTCACTCGATG |
|  | Cglac8-R (R4) | CTAGTCCAACGGGCCGTAAC |
|  | MiLRR-RLP1-F | ATGAAGCTCTTAGCGTTTTC |
|  | MiLRR-RLP1-R | CTAAACTAGAAAGTCCAAAT |
|  | Mi14-3-3-D1-F | ATGGCCAATGAAAGAGAGAA |
|  | Mi14-3-3-D1-R | TCACTCTTGGCCTTCACCAG |
| Bar-F (F3) | | TCAAATCTCGGTGACG |
| Bar-R (R3) | | ATGAGCCCAGAACGACGC |
| pUC57-F (F5) | | TCAGTTCGAGCTTTCCCACT |
| pUC57-R (R5) | | AAGGCACTCTTTGCTGCTTG |
|  | H852 (F1) | TTGTCCGTCAGGACATTGTT |
|  | H850 (R1) | AACTCACCGCGACGTCTGTC |
|  | Cglac8H-F (F2) | CAGGTGAGGCGAAGGTGCGTAG |
|  | Cglac8H-R (R2) | ATAGTCGTGGCCCGTGGAGGAG |
|  | qCglac8-F | CACCGTCCAGCAACAATATG |
|  | qCglac8-R | ATTCTTCTCCGCAAGCTTCA |
|  | qMiLRR-RLP1-F | TCCAGCTCTTTGCAACCTTT |
|  | qMiLRR-RLP1-R | GATTCTCCCCGAAAACTGGT |
|  | qMi14-3-3-D1-F | TCGATGAGCATTTGATTCCA |
|  | qMi14-3-3-D1-R | GGATGGGATGGGTAGGAGAT |
|  | Avr1b-PS-F | CGGAATTTTAATTAAGAATTCATGCGTCTATCTTTTGTGCTTTCTC |
|  | Avr1b-PS-R | CACTATAGGGAGAACCTCGAGTCAGCTCTGATACAGGTGAAAGGT |
|  | Cglac8-PS-F | CGGAATTTTAATTAAGAATTCATGGCTGCCTTCACTCGATG |
|  | Cglac8-PS-R | CACTATAGGGAGAACCTCGAGCTAGTCCAACGGGCCGTAACG |
|  | Cglac8^∆SP^-F | CGGAATTTTAATTAAGAATTCGCGCAGACGAGAAGATATAA |
|  | Cglac8^∆SP^-R | CACTATAGGGAGAACCTCGAGCTAGTCCAACGGGCCGTAACG |
|  | Cglac8-C-F | GACATCACCGCTAGCGAATTCATGGCTGCCTTCACTCGATG |
|  | Cglac8-C-R | TAGTCTGCTGGATCCGCGGCCGCCTAGTCCAACGGGCCGTAACG |
|  | Cglac8-BK-F | ATGGCCATGGAGGCCGAATTCATGGCTGCCTTCACTCGATG |
|  | Cglac8-BK-R | CCGCTGCAGGTCGACGGATCCCTAGTCCAACGGGCCGTAACG |
|  | Cglac8-EG-F | GCTGCGGCAGCGGCCGAATTCATGGCTGCCTTCACTCGATG |
|  | Cglac8-EG-R | TTATCTAGATCCGGTGGATCCCTAGTCCAACGGGCCGTAACG |
|  | LRR-AD-F | GCCATGGAGGCCAGTGAATTCATGAAGCTCTTAGCGTTTTCCCT |
|  | LRR-AD-R | CAGCTCGAGCTCGATGGATCCCTAAACTAGAAAGTCCAAATCAGAAACC |
|  | 1433-AD-F | GCCATGGAGGCCAGTGAATTCATGGCCAATGAAAGAGAGAACC |
|  | 1433-AD-R | CAGCTCGAGCTCGATGGATCCTCACTCTTGGCCTTCACCAGA |
|  | 1433-BK-F | ATGGCCATGGAGGCCGAATTCATGGCCAATGAAAGAGAGAACC |
|  | 1433-BK-R | CCGCTGCAGGTCGACGGATCCTCACTCTTGGCCTTCACCAGA |
|  | Cglac8-nY-F | CATTTAAATCTCGAGGGATCCATGGCTGCCTTCACTCGATG |
|  | Cglac8-nY-R | GGTGGCGATGGATCTTCTAGAGTCCAACGGGCCGTAACG |
|  | LRR-cY-F | GAGGAGGACCTGCTTTCTAGAATGAAGCTCTTAGCGTTTTCCCT |
|  | LRR-cY-R | CGCCGGACGGGTACCGGATCCAACTAGAAAGTCCAAATCAGAAACCTC |
|  | 1433-cY-F | GAGGAGGACCTGCTTTCTAGAATGGCCAATGAAAGAGAGAACC |
|  | 1433-cY-R | CGCCGGACGGGTACCGGATCCCTCTTGGCCTTCACCAGATTGA |
|  | 1433-nY-F | CATTTAAATCTCGAGGGATCCATGGCCAATGAAAGAGAGAACC |
|  | 1433-nY-R | GGTGGCGATGGATCTTCTAGACTCTTGGCCTTCACCAGATTGA |
|  | 8-Myc-F | GCCATGGCTGATATCGGATCCATGGCTGCCTTCACTCGATG |
|  | 8-Myc-R | TGGTGGTGCTCGAGTGCGGCCGCCTACAGGTCCTCCTCTGAGATCAGCTTCTGCTCCTCGTCCAACGGGCCGTAACG |
|  | LRR-Flag-F | GCCATGGCTGATATCGGATCCATGAAGCTCTTAGCGTTTTCCCT |
|  | LRR-Flag-R | TGGTGGTGCTCGAGTGCGGCCGCCTACTTGTCGTCATCGTCTTTGTAGTCAACTAGAAAGTCCAAATCAGAAACC |
|  | 1433-Flag-F | GCCATGGCTGATATCGGATCCATGGCCAATGAAAGAGAGAACC |
|  | 1433-Flag-R | TGGTGGTGCTCGAGTGCGGCCGCTCACTTGTCGTCATCGTCTTTGTAGTCCTCTTGGCCTTCACCAGA |
|  | 1433-Myc-F | GCCATGGCTGATATCGGATCCATGGCCAATGAAAGAGAGAACC |
|  | 1433-Myc-R | TGGTGGTGCTCGAGTGCGGCCGCTCACAGGTCCTCCTCTGAGATCAGCTTCTGCTCCTCCTCTTGGCCTTCACCAGA |
|  | LRR-PS-F | CGGAATTTTAATTAAGAATTCATGAAGCTCTTAGCGTTTTCCCT |
|  | LRR-PS-R | CACTATAGGGAGAACCTCGAGCTAAACTAGAAAGTCCAAATCAGAAACC |
|  | LRR^∆SP^-F | CGGAATTTTAATTAAGAATTCTCCAAAACTATCAAGCGTGACG |
|  | LRR^∆SP^-R | CACTATAGGGAGAACCTCGAGCTAAACTAGAAAGTCCAAATCAGAAACC |
|  | LRR-EG-F | GCTGCGGCAGCGGCCGAATTCATGAAGCTCTTAGCGTTTTCCCT |
|  | LRR-EG-R | TTATCTAGATCCGGTGGATCCCTAAACTAGAAAGTCCAAATCAGAAACC |
|  | 1433-EG-F | GCTGCGGCAGCGGCCGAATTCATGGCCAATGAAAGAGAGAACC |
|  | 1433-EG-R | TTATCTAGATCCGGTGGATCCTCACTCTTGGCCTTCACCAGA |
|  | MiActin-F | GTTTCCCAGTATTGTGGGTAGG |
|  | MiActin-R | AGATCTTTTCCATATCATCCCAGTT |
|  | qMiPR1-F | TCGCCCAAAACTATGCCAATCAA |
|  | qMiPR1-R | AGCCGATGAAGGTTCCACCGTTG |
|  | qMiACO-F | GGGAGCAAAAGTTCAACGAG |
|  | qMiACO-R | CTCCAGCTCCAGGTTCTCAC |
|  | qMiPDF1.2-F | GGTCAAGTGGGACATGGTCA |
|  | qMiPDF1.2-R | GAGCAGGGAACACATAGTTGC |
|  | AtActin-F | AAGCTCTCCTTTGTTGCTGTT |
|  | AtActin-R | GACTTCTGGGCATCTGAATCT |
|  | qAtPR1-F | AGGCTAACTACAACTACGCTGCG |
|  | qAtPR1-R | GCTTCTCGTTCACATAATTCCCAC |
|  | qAtACO-F | CCAGCTGGTAGCATCCACAA |
|  | qAtACO-R | ATCATTACCACGGCGACTCC |
|  | qAtPDF1.2-F | CCAAACATGGATCATGCAAC |
|  | qAtPDF1.2-R | CACACGATTTAGCACCAAAGA |
|  | SlActin-F | CGGTGACCACTTTCCGATCT |
|  | SlActin-R | TCCTCACCGTCAGCCATTTT |
|  | qSlPR1-F | ATCTCATTGTTACTCACTTGTC |
|  | qSlPR1-R | AACGAGCCCGACCA |
|  | qSlACO-F | GCGCCACTCTATTGTGGTTA |
|  | qSlACO-R | TGCATCACTTCCTGGATTGI |
|  | qSlPI-F | GAAGTAATTAAGCAGCCACAATATG |
|  | qSlPI-R | GCCCCCCTTATTTTTTCC |
|  | LRR-TRV2-F | GTGAGTAAGGTTACCGAATTCGTAACAATCATTTGGTGGGTACCA |
|  | LRR-TRV2-R | CGTGAGCTCGGTACCGGATCCCTTCAATGTACATTTCTTTCAAAAACC |
|  | 1433-TRV2-F | GTGAGTAAGGTTACCGAATTCATCGCCAAGCTCGCCGAG |
|  | 1433-TRV2-R | CGTGAGCTCGGTACCGGATCCGTCAGTGCAAATATTTGATAACTCACC |
